# The structure, biosynthesis, and biological roles of fetuin-A: A review

**DOI:** 10.3389/fcell.2022.945287

**Published:** 2022-07-18

**Authors:** Endeshaw Chekol Abebe, Zelalem Tilahun Muche, Awigchew Behaile T/Mariam, Teklie Mengie Ayele, Melaku Mekonnen Agidew, Muluken Teshome Azezew, Edgeit Abebe Zewde, Tadesse Asmamaw Dejenie, Misganaw Asmamaw Mengstie

**Affiliations:** ^1^ Department of Medical Biochemistry, College of Health Sciences, Debre Tabor University, Debre Tabor, Ethiopia; ^2^ Department of Physiology, College of Health Sciences, Debre Tabor University, Debre Tabor, Ethiopia; ^3^ Department of Pharmacy, College of Health Sciences, Debre Tabor University, Debre Tabor, Ethiopia; ^4^ Department of Medical Biochemistry, College of Medicine and Health Sciences, University of Gondar, Gondar, Ethiopia

**Keywords:** fetuin-A, structure, biosynthesis, biological roles, inflammation

## Abstract

Fetuin-A is a heterodimeric plasma glycoprotein containing an A-chain of 282 amino acids and a B-chain of 27 amino acid residues linked by a single inter-disulfide bond. It is predominantly expressed in embryonic cells and adult hepatocytes, and to a lesser extent in adipocytes and monocytes. Fetuin-A binds with a plethora of receptors and exhibits multifaceted physiological and pathological functions. It is involved in the regulation of calcium metabolism, osteogenesis, and the insulin signaling pathway. It also acts as an ectopic calcification inhibitor, protease inhibitor, inflammatory mediator, anti-inflammatory partner, atherogenic factor, and adipogenic factor, among other several moonlighting functions. Fetuin-A has also been demonstrated to play a crucial role in the pathogenesis of several disorders. This review mainly focuses on the structure, synthesis, and biological roles of fetuin-A. Information was gathered manually from various journals *via* electronic searches using PubMed, Google Scholar, HINARI, and Cochrane Library from inception to 2022. Studies written in English and cohort, case-control, cross-sectional, or experimental studies were considered in the review, otherwise excluded.

## Introduction

Fetuin-A, formerly known as phosphoprotein 63 KDa (pp63) or countertrypin, is a plasma carrier protein that helps to transport and make a wide range of substances available in the bloodstream. It is a negatively charged glycoprotein circulating in the blood and extracellular fluid with several days of half-life (Kettler et al., 2003; Wojtysiak-Duma et al., 2010). The molecular weight of fetuin-A ranges from 51 to 67 kDa, depending on the degree of glycosylation (Brown et al., 1992). According to numerous studies, fetuin-A is considered a multifunctional protein that participates in myriads of essential biological activities, such as the regulation of bone and calcium metabolism and the insulin signaling pathway. Besides, it acts as a protease inhibitor, inflammatory mediator, anti-inflammatory partner, atherogenic factor, and adipogenic factor, among several other moonlighting functions. Fetuin-A has also been found to have a key role in the development of various clinical conditions, such as insulin resistance (IR), type 2 diabetes mellitus (T2DM), metabolic disorders, nonalcoholic fatty liver disease (NAFLD), cardiovascular diseases (CVDs), tumors, and brain disorders (Kettler et al., 2003; Wojtysiak-Duma et al., 2010; Mori et al., 2012; Sardana et al., 2021). This review article primarily aimed to explore the structure, biosynthesis, and biological roles of fetuin-A.

Information was manually retrieved from PubMed, Google Scholar, HINARI, and Cochrane Library from inception to 2022. The search was done using search terms: fetuin-A, alpha2 Heremans-Schmid Glycoprotein, fetuin-A structure, function, fetuin-B structure, function, the role of fetuin-A in insulin signaling, bone metabolism, the inflammation, protease regulation. Besides, the adipogenic role, the atherogenic role, and opsonizing properties of fetuin-A. The role of fetuin-A in the tumor, NAFLD, CVDs, brain development, and lesions. Those articles written in English and which were cross-sectional, cohort, case-control, or experimental studies were included in the study. However, articles written in languages other than English were excluded from the review. As this review primarily focused on the physiological roles of fetuin-A, some articles detailing the clinical disorders associated with fetuin-A were also not included. Moreover, articles about the clinical implications of fetuin-A, such as diagnostic and therapeutic roles, are outside the scope of this review and were excluded.

## Overview of the fetuin family

Fetuins are a family of a large group of proteins belonging to the cystatin superfamily of cysteine protease inhibitors of the metalloprotease, papain, calpain, cathepsin, and caspase families, possessing a domain of conserved cysteine residues responsible for their protease inhibitory activity (Sardana et al., 2021). The cystatin superfamily comprises type 1 (mainly intracellular proteins), type 2 (mainly extracellular proteins), type 3 (plasma proteins), and unclassified cystatins (Jahnen-Dechent et al., 2011). The fetuin family is members of four structurally related type 3 cystatins. Along with the fetuin family, cystatins, histidine-rich glycoproteins, and kininogens are grouped under the type 3 cystatin family (Brown and Dziegielewska, 1997). The fetuin family constitutes two paralogue proteins, which are homologous proteins encoded by duplicated genes, known as fetuin-A and fetuin-B (Olivier et al., 2000; Sardana et al., 2021).

A group of orthologous plasma proteins referred to as fetuin-A was isolated long years ago in different animals, including humans, sheep, pigs, cows, reptiles, fish, birds, marsupials, gerbils, rats, mice, and rodents. The first discovery of fetuin-A dates back to 1944 by K. Pedersen in the blood of a fetal calf (hence the name “fetuin”) (Lin et al., 2018). Many years later, the independent studies by J.F. Heremans and K. Schmid with W. Burgi in 1960 and 1961, respectively, purified fetuin-A in humans, and hence it is now also known as α2-Heremans-Schmid glycoprotein (AHSG) in recognition of the discoverers. The comigration of fetuin-A with the α2-globulin fraction of plasma proteins during electrophoresis provides the prefix “α2” in its official name, AHSG (Jahnen-Dechent et al., 2011). Fifty-five years later, in 1999, Olivier et al. discovered the second member of the mammalian fetuin family and the paralog of fetuin-A in rats, mice, and humans, designated as fetuin-B (Olivier et al., 2000).

Fetuin-A and fetuin-B were demonstrated to have a similar evolutionary origin and close chromosomal locations within the vertebrate genomes. In humans, both are encoded from duplicated genes which are still co-localized side by side in a single chromosomal area (3q27). Their encoding genes are thought to have evolved phylogenetically from cystatin *via* the process of gene duplication and gene segment exchange (Pedersen, 1944; Olivier et al., 2000; Floehr et al., 2016). Moreover, fetuin-A and fetuin-B have structural similarities, such as domain homology and conserved cysteine residues. Both fetuins contain three domains (D1, D2, and D3), identical numbers and locations of all C-residues, as well as an identical short LETXCHXL motif (Olivier et al., 2000). Unlike other members of the cystatin superfamily, fetuins lack cystatin activity due to the mutation and loss of relevant sequence motifs (G residue, QXVXG motif, and PW dipeptide) that are required to activate the cystatin inhibitory domain and exhibit cystatin protease activity (Dziegielewska and Brown, 1995; Olivier et al., 2000). However, they exhibit significant differences in amino acid sequences as well as other important residues/motifs, as summarized in Table 1.

**TABLE 1:** Comparisons between human fetuin-A and fetuin-B.

Features	Fetuin A	Fetuin B	References
Discovery	In 1944 by K. Pedersen	In 1999 by Olivier et al	Pedersen. (1944); Olivier et al. (2000)
Tissue distribution	Hepatocytes and embryonic cells	Hepatocytes and placenta	Olivier et al. (2000); Kettler et al. (2003); Stefan et al. (2006a)
Serum concentration	More abundant (300–1000 μg/ml)	Less abundant	Olivier et al. (2000); Kaushik et al. (2009); Singh et al. (2012); Floehr et al. (2016)
Encoding gene	AHSG gene in 3q27	FETUB gene in 3q27	Pedersen. (1944); Olivier et al. (2000)
Molecular weight	51-67KDa	42-60KDa	Brown et al. (1992); Olivier et al. (2000); Kettler et al. (2003); Wojtysiak-Duma et al. (2010); Karmilin et al. (2019)
Number of amino acids (including SS)	367	382	Olivier et al. (2000); Mori et al. (2011)
Structural domains	Possesses three domains (D1, D2, D3)	Contains the three domains, identical number and location of all C-residues; identical short LETXCHXL motif as in fetuin-A	Olivier et al. (2000); Mori et al. (2012); Komsa-Penkova et al. (2017)
DPTP tetrapeptide	Present and serve as a signature of fetuin-A	Absent	Olivier et al. (2000)
Calcium binding motif	Found in D1 domain	Absent	Olivier et al. (2000)
Connecting peptide	Found between A- and B-chain	Absent	Olivier et al. (2000)
Kunitz-type motif	D2 of fetuin-A harbors active Kunitz-type inhibitory site	Both the D1 and D2 of fetuin-B contain Kunitz-type motif	Olivier et al. (2000)
Cystatin activity	Specific amino acid residues activating cystatin inhibitory domain are partly lost and hence no cystatin activity	Most likely absent due to lack of these amino acid residues	Dziegielewska and Brown. (1995); Olivier et al. (2000)
Endogenous inhibitory activities	TGF-β, BMP, meprin-α and-β	Ovastacin, meprin-β	Dietzel et al. (2013); Cerdà‐Costa and Xavier Gomis‐Rüth. (2014); Stöcker et al. (2014); Cuppari et al. (2019); Karmilin et al. (2019); Afrisham et al. (2020)
Function	Serves as APP and takes part in osteogenesis, calcium metabolism, insulin signaling, endocytosis, and brain growth	Act as APP and involved in bone metabolism and fertilization	Denecke et al. (2003); Mori et al. (2011); Dietzel et al. (2013); Cerdà‐Costa and Xavier Gomis‐Rüth. (2014); Stöcker et al. (2014)

Abbreviations: AHSG, α2-Heremans-Schmid glycoprotein; APP, acute phase protein; BMP, bone morphogenetic protein; DPTP, Aspartate-proline-threonine-proline tetrapeptide; TGF, transforming growth factor; SS, signal sequence.

In addition, these paralogue proteins are found to have similar biological functions and both serve as acute-phase proteins (APP) as well as regulators of insulin signaling and bone metabolism, but they differ in many other biological functions. Fetuin-A has a well-established broad range of functions in humans, such as regulation of osteogenesis, calcium metabolism, endocytosis, and brain growth, among many other moonlighting functions (Denecke et al., 2003). On the other hand, fetuin-B has a crucial role in fertilization by blocking the proteolytic activity of ovastacin, which is a member of the astacin family that mediates the hardening of the zona pellucida (Dietzel et al., 2013; Cerdà Costa and Xavier Gomis Rüth, 2014; Stöcker et al., 2014).

Furthermore, fetuin-A and fetuin-B are closely related with regard to their site of synthesis and tissue distribution. Fetuin-A is secreted largely by the liver in adults, but it is extensively expressed by multiple tissues such as the liver, kidney, brain, choroid plexus, skin, and gastrointestinal tract in the fetus (Kettler et al., 2003; Stefan et al., 2006a). Blood fetuin-A is found in relatively high concentrations throughout life, with substantially higher levels in fetuses than in adults. According to a large cohort study of neonates, blood levels of fetuin-A are highest in very low birth weight newborns between 24 and 30 completed weeks of gestational age, with levels 2–3 times greater than those observed in term birth. Fetuin-A levels gradually declined with intrauterine as well as extrauterine maturation, reaching adult levels at about 37 completed weeks of pregnancy (Häusler et al., 2009). Serum levels of fetuin-A normally range between 300 and 1000 μg/ml in both sexes of healthy adult humans, though it is a 5–50 fold more abundant in fetal blood (Kaushik et al., 2009; Singh et al., 2012). These development-associated changes in fetuin levels, however, are known to vary substantially between species (Dziegielewska and Brown, 1995). Concerning fetuin-B, it is also predominantly produced by hepatocytes and, to a lesser extent, by many secretory tissues, including the placenta. Quantitatively, fetuin-B is found to be less plentiful in the blood than fetuin-A (Floehr et al., 2016). A study by Olivier et al. (2000)indicated that the expression level of fetuin-A and fetuin-B in hepatocytes is in the ratio of eight to one, as demonstrated in adult rats.

## Structure and biosynthesis of fetuin-A

**FIGURE 1:**
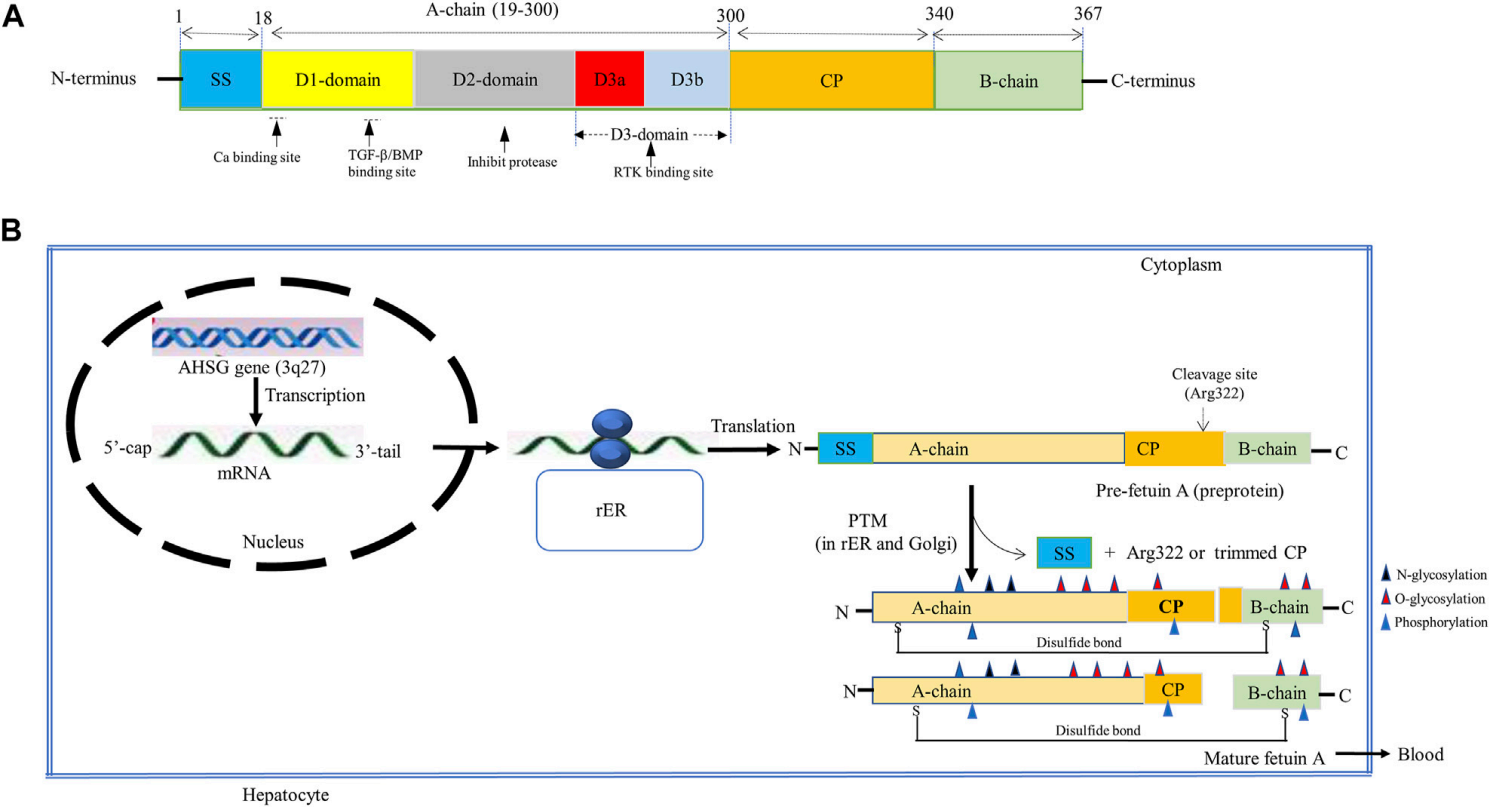
Schematic representation of **(A)** Fetuin-A structure. The pre-fetuin-A contains 367 amino acids, including 18 amino acid size SS (Pedersen, 1944; Brown et al., 1992; Dziegielewska and Brown, 1995; Brown and Dziegielewska, 1997; Olivier et al., 2000; Denecke et al., 2003; Kettler et al., 2003; Stefan et al., 2006a; Häusler et al., 2009; Wojtysiak-Duma et al., 2010; Jahnen-Dechent et al., 2011; Mori et al., 2012; Dietzel et al., 2013; Cerdà‐Costa and Xavier Gomis‐Rüth, 2014; Stöcker et al., 2014; Floehr et al., 2016; Lin et al., 2018; Sardana et al., 2021); 282 amino acids A-chain (19–300), 40 amino acids CP (301–340), and 27 amino acids long B-chain (341–367). A-chain is composed of two cystatin-like domains (D1 and D2) and a variable non-cystatin domain, D3 (D3a and D3b). Functionally, the D1 provides binding sites for calcium, TGF- β, and BMP, the D2 inhibits cysteine protease and D3 interacts with the insulin receptor. **(B)** Fetuin-A biosynthesis. Fetuin-A is encoded by the AHSG gene located on the 3q27 locus that produces a single copy of mRNA encoding a pre-fetuin-A. The pre-fetuin-A consisting of SS, A-chain, CP, and A-chain undergoes PTMs (glycosylation, proteolysis, folding, and phosphorylation). Glycosylation takes place at Asp residues (N-glycosylation), Thr and Ser residues (O-glycosylation). Then SS and Arg322 will be removed through proteolysis with certain unknown proteinase to form a mature fetuin-A containing a full length of CP apart from Arg322 or C-terminally trimmed CP, interlinked by a disulfide bond formed between Cyst-14 and Cys-340. Then phosphorylation at multiple Ser and Thr residues, mostly in plasma, occurs by FAM20C. Abbreviations: AHSG, α2-Heremans-Schmid glycoprotein; BMP, Bone Morphogenic protein; CP, connecting peptide; FAM20C, family with sequence similarity 20 member C; PTMs, post-translational modifications; rER, rough endoplasmic reticulum; RTK, receptor tyrosine kinase; SS, signal sequence; TGF- β, Transforming growth factor-beta.

Fetuin-A is a hepatokine that is predominantly (more than 95%) synthesized in the adult liver, with the remaining lesser extent produced in adipocytes, monocytes/macrophages, and other cells (Stefan et al., 2006a; Wojtysiak-Duma et al., 2010; Chatterjee et al., 2013). It is a heterodimeric globular protein that is encoded by the AHSG gene located on the 3q27 human chromosome locus, containing seven exons and six introns of nearly 8.2 kb long in length (Mori et al., 2011). The transcription of this gene produces a single copy of mRNA that encodes a single chain preprotein of human fetuin-A containing 367 amino acids (Osawa et al., 1997; Reinehr and Roth, 2008; Kaushik et al., 2009). The process of transcription is regulated by several CCAAT enhancer-binding proteins (C/EBP)-β and nuclear factor (NF)-1 binding sites in the promoter region (Falquerho et al., 1992; Banine et al., 2000). The pre-fetuin-A consists of two polypeptide chains, namely the heavy A-chain, which was first demonstrated by Yoshioka et al. in 1986, and the light B-chain, which was first reported by Gejyo et al. in 1983 (Gejyo et al., 1983; Yoshioka et al., 1986). While the A-chain is a larger polypeptide chain possessing 282 amino acid residues, the B-chain is a smaller chain consisting of 27 amino acids (Osawa et al., 1997). This precursor protein also encompasses an additional 18 amino acid containing signal sequence (SS) in N-terminus and a 40 amino acid long connecting peptide (CP) in between the two chains (Figure 1A) (Lee et al., 1987; Osawa et al., 1997).

Although posttranslational modification (PTM) of fetuin-A is less clearly understood and variable, a plethora of studies indicated that both A and B polypeptide chains of human fetuin-A undergo PTMs involving glycosylation, proteolytic cleavage, folding, and phosphorylation to form active fetuin-A (Figure 1B) (Haglund et al., 2001; Wilson et al., 2002; Kübler et al., 2007; Dabrowska et al., 2015). A study by Lin et al. (2018) showed that the modification of native fetuin-A frequently begins with N-glycosylation in the rough endoplasmic reticulum followed by the O-glycosylation in the Golgi apparatus to add up to 23.6% of carbohydrate. N-linked glycosylation is identified to take place at asparagine (Asn) residues (Asn156 and Asn176) of the A chain, whereas O-linked glycosylation may occur at threonine (Thr) residues (Thr270, Thr339, and Thr 342) and serine (Ser) residues (Ser280, Ser293, and Ser346) of the A and B chains (Gejyo et al., 1983; Yoshioka et al., 1986). The glycosylated fetuin-A then has been demonstrated to undergo proteolytic processing and phosphorylation (Haglund et al., 2001; Lin et al., 2018). Proteolytic processing of preprotein involves removal of SS from the N-terminus and cleavage of C-terminal arginine at position 322 with a certain unknown proteinase, forming an opening between arginine-322 and threonine-323 of the two polypeptide chains. The full length of CP other than arginine-322 or trace C-terminally trimmed CP fragments of various degrees could be formed. A 40-amino acid containing CP has been reported to be completely cleaved off in abnormal conditions *s*uch as sepsis. Thus, modified fetuin-A proved to possess an A-chain between 1–282 amino acids, CP from 283–321 residues, and a B-chain between 323–349 amino acid residues (Kellermann et al., 1989; Haglund et al., 2001; Lin et al., 2018; Ochieng et al., 2018).

Subsequently, modified A- and B-chains undergo proper folding and are held together by a single interchain disulfide bridge to form a heterodimeric, three-dimensional mature glycoprotein. Indeed, there are six disulfide bridges within fetuin-A, but only a disulfide bond formed between cysteine-14 and cysteine-340 links the A-chain and the B-chain (Dabrowska et al., 2015). Fetuin-A is eventually released into the bloodstream from the liver, although it is influenced by many positive and negative factors (Icer and Yıldıran, 2020). Endogenous factors such as increased blood glucose and free fatty acid (FFA) levels have been established to enhance fetuin-A release. While high blood glucose levels induce fetuin-A release by stimulating extracellular signal-regulated kinase 1/2, increased blood FFA promotes fetuin-A secretion by increasing NF-_K_B activity (An et al., 2012; Icer and Yıldıran, 2020). It is evident from the increased expression of fetuin-A in fatty livers and its decreased levels in people who had less liver fat. High-fat diet-fed animals and obese diabetic patients are associated with increased plasma levels of fetuin-A, indicating FFA enhances the secretion of fetuin-A from the liver (Lin et al., 1998; Kelley et al., 2003; Stefan et al., 2006b).

In addition, several prior studies suggested that low and high dietary energy intake may affect serum fetuin-A levels (Choi et al., 2013; Samocha-Bonet et al., 2014). Exogenous factors like curcumin, resveratrol, dairy products, niacin, alcohol, and coffee consumption have all been demonstrated in studies to be negative factors that decrease fetuin-A release and its blood concentrations (Kaushik et al., 2009; An et al., 2012; Öner-İyidoğan et al., 2013; Ghaffari et al., 2017; Mukhuty et al., 2017). On the other hand, dietary intake of omega-3 fatty acids has been shown to increase hepatic fetuin-A secretion (An et al., 2012). The mechanism of how several of these factors affect fetuin-A release has not yet been clearly understood, but some of them have postulated mechanisms. For instance, curcumin has been shown to reduce fetuin-A expression by regulating peroxisome proliferator-activated receptor-γ (PPAR-γ) and stimulating AMP-activated kinase (AMPK) (Ochi et al., 2014; Seyithanoğlu et al., 2016). Dietary niacin intake lowers blood lipids that negatively affect fetuin-A expression (Andersen et al., 2008). In contrast, Kaushik et al. (2009) indicated that dietary niacin may enhance fetuin-A expression by increasing endogenous glucocorticoids, showing the need for further confirmatory studies. The accumulated body of data confirmed that increased dietary intake of FFA can positively affect fetuin-A expression by elevating blood levels of FFA (Okamoto et al., 2018).

In the blood circulation, secreted fetuin-A will undergo phosphorylation at several phosphosites to form phosphorylated fetuin-A (Haglund et al., 2001). Studies done on hepatoma cell lines (HepG2) demonstrated multiple phosphorylations at serine residues (Ser134, Ser135, Ser138, Ser 325, Ser328, and Ser330) and threonine residue (Thr319) by the family with sequence similarity 20 member C (FAM20C) kinase (Falquerho et al., 1992; Haglund et al., 2001; Matsui et al., 2009). It has been shown that up to 20% of human plasma fetuin-A is phosphorylated at Ser330 (Stirnberg et al., 2015). Phosphorylated fetuin-A seems physiologically important to counteract both the insulin signaling pathway and ectopic calcification (Matsui et al., 2009). Overall, the PTM of fetuin-A is thought to be responsible for its extensive range of functions identified in the past few decades.

The A-chain of mature fetuin-A is composed of two homologous, tandemly arranged cystatin-like domains, D1 and D2. These two domains contain 116–118 amino acid residues located in the N-terminal end and form α-helical structure, beta-pleated sheet, and reverse turns, accounting for 29, 24, and 26%, respectively (Mori et al., 2012; Komsa-Penkova et al., 2017). The D1 and D2 of most fetuin-A sequences contain two dibasic motifs, db1 and db2, respectively, that are responsible for the trypsin susceptibility of fetuin-A (Olivier et al., 2000). While the D1 domain is a well-studied domain with binding motifs for hydroxyapatite and transforming growth factor-β (TGF- β) superfamily in its beta sheet, the D2 domain is still not well characterized, but it has been proposed to inhibit cysteine protease in the rats (Komsa-Penkova et al., 2017). The third domain found in the A-chain, which is less conserved (or variable) between species and has a unique histidine-rich C-terminal domain, is known as D3. Although it lacks sequence homology to cystatins, the D3 domain has been suggested to have a high affinity for the insulin receptor. It can be classified as the D3a subdomain, which is a hydrophobic, proline-rich subunit found in the N-terminal region, and the D3b subdomain, which is found in the C-terminal end, consisting of a connecting peptide (Brown et al., 1992; Brown et al., 1992). On the other hand, the B chain is a short polypeptide chain made up of 27 amino acid residues and may undergo PTMs like O-linked and N-linked glycosylation (Brown et al., 1992; Singh et al., 2012).

## Biological roles of fetuin-A

Reports from a plethora of evidence indicate that fetuin-A is a multifunctional protein that plays a wide array of important physiological and pathological roles. (Wojtysiak-Duma et al., 2010; Brown and Dziegielewska, 1997; Pedersen, 1944; Dietzel et al., 2013; Mori et al., 2012). This is achieved by binding with multiple receptors, such as insulin, growth hormone, growth factors [nerve growth factor (NGF), platelet-derived growth factor (PDGF), transforming growth factor (TGF)-II, TGF-β2 and basic fibroblast growth factor (bFGF)], and a number of toll-like (TLR) receptors (Nie, 1992; Jahnen-Dechent et al., 2011; Pal et al., 2012). In the last few decades, the number of biological functions attributed to fetuin-A has increased exponentially. It has been identified to take part in a myriad of important biological activities, such as regulation of bone remodeling (osteogenesis and bone resorption) and calcium metabolism, insulin signaling, endocytosis, brain development, and protein metabolism. Besides, fetuin-A is involved in varieties of anti-inflammatory and inflammatory attributes, neutrophil and platelet degranulation, lymphocyte stimulation, keratinocyte migration, and carriers of metals and small molecules in the bloodstream by binding with fatty acids, thyroid hormones, phosphate, and calcium ions (Dabrowska et al., 2015; Lin et al., 2018). Furthermore, fetuin-A plays a key role in the pathogenesis of several metabolic disorders such as IR, T2DM, NAFLD, CVDs, and autoimmune disorders; breast tumor cell proliferative signaling; brain disorders such as ischemic stroke and neurodegenerative disease and more recently, psoriasis (Kettler et al., 2003; Wojtysiak-Duma et al., 2010; Mori et al., 2012; Sardana et al., 2021; Abebe et al., 2022). This part of the review comprehensively explores various biological roles of fetuin-A, with special emphasis on its physiological roles.

### Regulatory roles of fetuin-A in bone and calcium metabolism

Fetuin-A acts as a plasma carrier protein for calcium and phosphate to exquisitely regulate their levels in extracellular fluid. It represents a major proportion (25%) of the non-collagenous bone proteins found in bones and teeth (Jahnen-Dechent et al., 2011). Compared to other plasma proteins, fetuin-A is 300-fold more abundant in the adult and fetal bone matrix. The bone level of fetuin-A, however, progressively declines from childhood to maturity. Its levels are seven times and ten times, respectively, higher in neonatal bone and fetal bone than in adult bone (Lee et al., 1987; Jahnen-Dechent et al., 2011).

Recently, fetuin-A has been established as a negative regulator of bone and calcium metabolism (Binkert et al., 1999; Swallow et al., 2004). It serves as a potent local and systemic endogenous inhibitor of pathological calcification and mineralization, which is indeed associated with an increased risk of different cardiovascular morbidities (Heiss et al., 2003; Kettler et al., 2003). In mice devoid of fetuin-A, aberrant systemic ectopic calcification of soft tissues was seen, demonstrating the regulating role of fetuin-A on undesired calcification and bone osteogenesis (Jahnen-Dechent et al., 2011). This inhibitory action on extraosseous calcification is mediated by the binding of fetuin-A with insoluble calcium phosphate (or hydroxyapatite) and forming highly soluble, inactive, and stable colloidal fetuin-A-mineral complexes in the circulation called calciprotein particles (Heiss et al., 2003). Fetuin-A is responsible for the inhibition of about half of the calcium and phosphorus precipitation, implying that it is a major serum agent preventing vascular calcification. Thus, fetuin-A functions as a mineral chaperone, which is a carrier protein that aids in the transportation and clearance of potentially proinflammatory and procalcific cargo (Jahnen-Dechent et al., 2011).

A growing body of evidence proposes that the clustered, strongly negatively charged acidic amino acids (motif) in the D1 domain of fetuin-A have high-affinity binding sites for calcium-rich minerals and TGF-β superfamily (Kübler et al., 2007). Calciprotein particle prevents calcium salt precipitation, vascular calcification, and thus CVD by counteracting the actions of the TGF-β superfamily, such as TGF-β and bone morphogenetic protein (BMP), which are required for bone mineralization, as well as by acting as vacuum cleaners to remove precipitated calcium in extraosseous areas of the bone (Heiss et al., 2003; Swallow et al., 2004). Notably, fetuin-A only prevents the *de novo* formation of calcium phosphate (mineralization), not the dissolution of preformed minerals (Jahnen-Dechent et al., 2011). It does not interfere with the formation of mineral nuclei; rather, it acts as a shield for spontaneously formed mineral nuclei and keeps calciprotein particles stable. Fetuin-A, on the other hand, prevents mineral nuclei from growing and aggregating into larger entities, curbing mineral precipitation (Rochette et al., 2009).

Moreover, fetuin-A abrogates vascular calcification by directly binding to the vascular smooth muscle cells and thereby limits oxidative stress, inflammation, and vascular damage (Jahnen-Dechent et al., 2011). This is largely achieved by inhibiting apoptosis and caspase cleavage (Jahnen-Dechent et al., 2011). Fetuin-A is taken up by vascular smooth muscle cells and concentrated in intracellular vesicles. Then, through secretory vesicles, fetuin-A is released from both apoptotic and viable vascular smooth muscle cells. Consequently, fetuin-A in vesicles rendered them incapable of nucleating calcium phosphate precipitation. Fetuin-A also enhanced phagocytosis of vesicles by vascular smooth muscle cells. Hence, the uptake of fetuin-A by cells and the vesicular recycling of calciprotein particles decrease both apoptosis and calcification of these cells (Reynolds et al., 2004). Altogether, fetuin-A protects against aberrant calcification by stabilizing mineral complexes in the calciprotein particles and mediating their transport and clearance.

Low levels of fetuin-A, on the other hand, have been found to increase the propensity for systemic vascular calcification and cardiovascular morbidities (Jahnen-Dechent et al., 2011). Patients with different heart diseases, such as coronary artery disease, ischemic cardiomyopathy, and aortic stenosis, have lower levels of fetuin-A. An inverse correlation has also been found between fetuin-A concentrations and calcified coronary artery disease, showing the vascular calcification inhibitory function of fetuin-A (Mori et al., 2012). Moreover, severe abnormalities in fetuin-A levels occur in chronic kidney disease (CKD) and are correlated with the mineral bone disorder. Caglar et al. (2008) found that circulating fetuin-A levels drop dramatically as CKD progresses, and fetuin-A deficiency is linked with an increased propensity for calcification and a poor prognosis in CKD patients. This has recently been confirmed by another study demonstrating that fetuin-A (especially those derived from the kidney) locally safeguards the kidney from hypoxia-induced renal damage and functional deterioration by acting as a potent mineral scavenger. It plays a salient role in binding and clearance of accumulated calcium mineralized matrix in fetal hypoxic kidney and acting as an ectopic calcification inhibitor protecting the integrity of kidney tissues and preventing the progression to CKD (Rudloff et al., 2021).

### Inflammatory and anti-inflammatory roles of fetuin-A

Fetuin-A has a very complex role in modulating inflammatory responses, with increasing evidence pointing to both pro-inflammatory and anti-inflammatory attributes (Mukhopadhyay et al., 2014). Even though it is still not clear which effect is dominant, fetuin-A seems to have both anti-inflammatory and inflammatory activity depending on its mode of activation (stimulus) in various clinical conditions (Komsa-Penkova et al., 2017). It is well established that fetuin-A is a negative acute-phase protein that is quickly lowered in response to acute inflammation (Ombrellino et al., 2001). Several studies have shown an inverse relationship between the serum level of fetuin-A and C-reactive protein (CRP), with fetuin-A levels decreasing and CRP levels rising following an inflammatory response (Kettler et al., 2003; Chen et al., 2009; Wojtysiak-Duma et al., 2010). The mechanism by which the serum level of fetuin-A decreases is more likely multifactorial, such as reduced hepatic production in response to inflammatory cytokines, increased excretion, or enhanced breakdown (Dabrowska et al., 2015). More importantly, the inflammatory cytokines released by the plethora of TLR (TLR2, TLR4, and TLR9) mediated immune responses have an inhibitory effect on hepatic fetuin-A synthesis (Mukhopadhyay et al., 2014).

Thus, fetuin-A has been shown to exhibit anti-inflammatory properties that serve as a protective agent against lethal systemic inflammation (Mori et al., 2012). This has been reported to have a beneficial role against various clinical conditions, like sepsis, endotoxemia, injury, chronic obstructive pulmonary disease (COPD), Crohn’s disease, ulcerative colitis, rheumatoid arthritis, pancreatitis, CKD, and brain pathologies (Ombrellino et al., 2001; Sato et al., 2007; Metry et al., 2008; Kuśnierz-Cabala et al., 2010; Wang et al., 2010; Wang and E Sama, 2012; Mukhopadhyay et al., 2014). The anti-inflammatory role of fetuin-A is possibly due to its direct antagonist role on TGF-β and tumor necrosis factor-alpha (TNF-α) mediated inflammation, as well as fetuin-A mediated inhibition of pathogen-associated molecular pattern (PAMP) induced release of high mobility group box protein 1 (HMGBP1) by innate immune cells. Fetuin-A has a protective role against inflammatory bowel disease (IBD) by blocking a zinc metalloproteinase (known as meprin-α), which is a key player in the development of IBD by stimulating inflammatory cytokines, as well as by protecting against intestinal inflammation by inhibiting HMGB1 release (Li et al., 2011). In addition, recent studies have convincingly demonstrated the role of decreased fetuin-A concentration in the pathogenesis of different brain pathologies, including cerebral ischemic injury and neurodegenerative diseases (multiple sclerosis and Alzheimer’s disease), indicating its anti-inflammatory attributes (Laughlin et al., 2014). This is reinforced by the fact that exogenous administration of fetuin-A attenuates neuronal inflammation and provides neuroprotection (Wang et al., 2010). Fetuin-A also exerts anti-inflammatory activities by a polyamine (known as spermines) and protects against brain lesions by suppressing inflammatory cytokine release (Wang and E Sama, 2012).

Moreover, a recent report by Rudloff et al. (2021) has elucidated that fetuin-A blocks molecular mechanisms linking fetal hypoxia and progression to renal fibrosis and inflammation in adults, thereby preserving kidney function. In contrast to the liver-derived fetuin-A, which is produced for systemic use, the kidney-derived fetuin-A has been demonstrated to play a local role with no systemic relevance. The kidney-derived fetuin-A has been identified as a hypoxia-inducible transcription factor (HIF) target gene, which locally safeguards the integrity of kidneys from hypoxia-induced chronic and progressive renal damage. Fetuin-A has been observed to possess evolutionarily conserved putative HIF-binding sites overlapping with its enhancer regions that increase the expression of fetuin-A in proximal kidney tubules in hypoxic conditions. It attenuates hypoxia-induced renal fibrosis and inflammation by employing several mechanisms, such as counteracting TGF-β1 and modulating macrophage polarization, in addition to antagonizing local calcification as discussed in the earlier section of this review. Fetuin-A inhibits the expression of fibrosis markers that cause renal fibrosis by counteracting TGF-β signaling. This has been reaffirmed by the antagonizing role of fetuin-A supplementation on the expression of fibrotic markers and renal tissue remodeling upon ischemia-reperfusion injury (Rudloff et al., 2021). This supports prior findings indicating fetuin-A as a soluble decoy receptor mimicking the TGF-β type II receptor and cytokine antagonist (Demetriou et al., 1996; Szweras et al., 2002). Fetuin-A also plays anti-inflammatory activities to protect the kidney against renal fibrosis by mitigating hypoxia-induced renal infiltration and polarization of pro-inflammatory M1 macrophages (Rudloff et al., 2021).

Fetuin-A has also been found as a positive acute phase reactant that increases in blood circulation in response to inflammation (Mukhopadhyay et al., 2014). This is supported by the study showing a significant positive relationship between fetuin-A and CRP, indicating the inflammatory activity of fetuin-A (Borsky et al., 2021). Different clinical studies revealed the pro-inflammatory effects of fetuin-A by demonstrating increased levels of fetuin-A in patients with metabolic syndrome, IR, T2DM, obesity, early-stage atherosclerosis, CVD such as coronary artery disease and peripheral artery disease, and NAFLD (Mathews et al., 2002; Stefan et al., 2006a; Mori et al., 2006; Sardana et al., 2021). Fetuin-A is not only a hepatokine but also an adipokine secreted from adipocytes, and its expression is directly linked to the amount of fat in adipocytes. In hyperlipidemic conditions, FFA upregulates fetuin-A expression, which occurs by stimulating its promotor activity through a transcription factor known as nuclear factor κB (NFκB) (Lin et al., 1998; Kelley et al., 2003; Stefan et al., 2006b; Das et al., 2021). But lipid-induced NF-κB-mediated fetuin-A promoter activation is not associated with C/EBP-β as demonstrated by Dasgupta et al. (2010) FFA fails to trigger fetuin-A expression in NF-*κ*B-knockout HepG2 cells, while it substantially enhanced fetuin-A levels in the cells injected with NF-κB, likely via the stimulatory effect of NF-κB on the promoter region of fetuin-A other than C/EBP-β.

**FIGURE 2:**
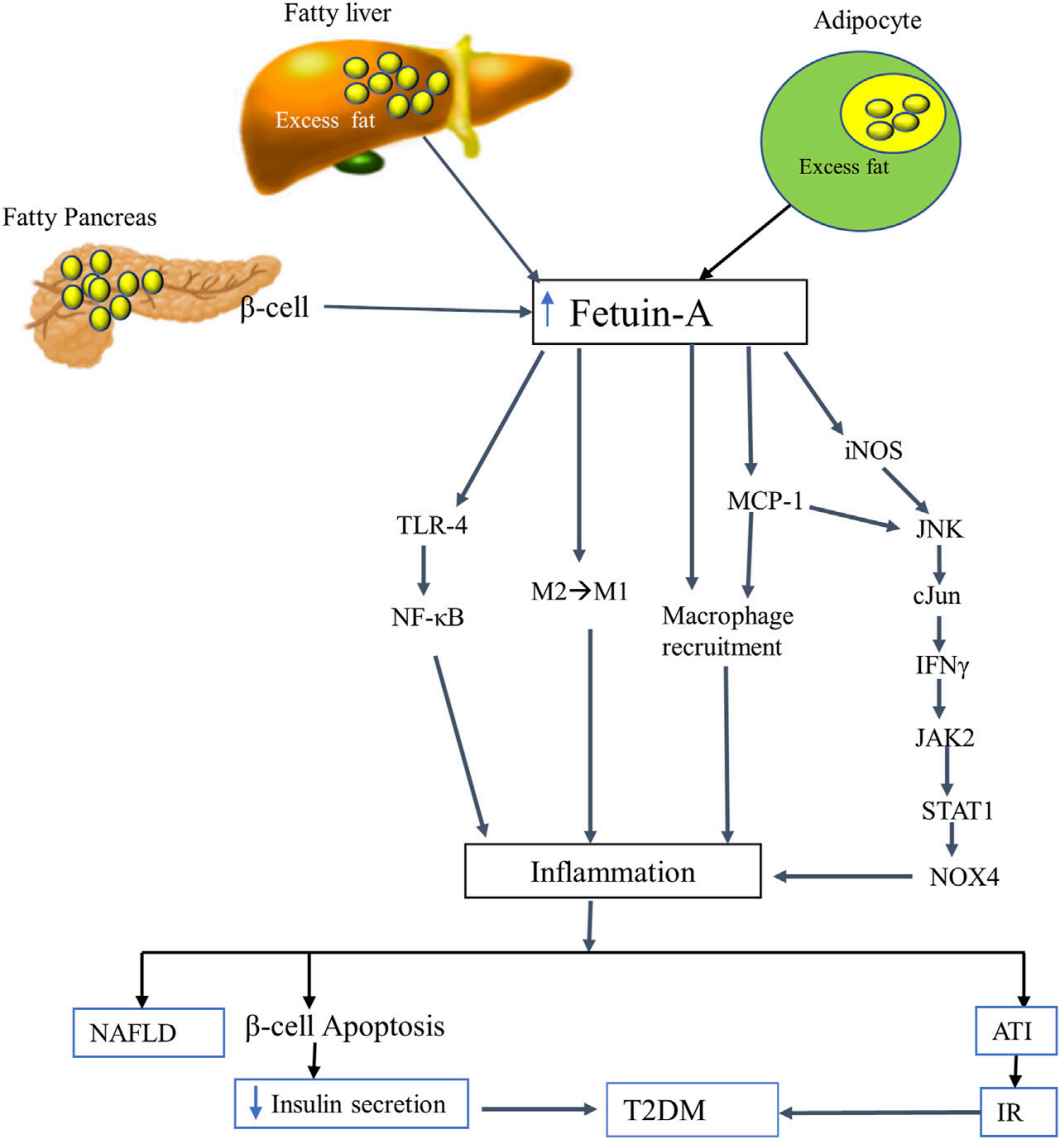
The putative inflammatory role of fetuin-A. FFA-mediated oversecretion of fetuin-A from the liver, adipocytes, and pancreatic beta cells induces an inflammatory signaling pathways by employing several mechanisms. Fetuin-A acts as a ligand for TLR-4 and a macrophage-polarizing agent that transforms the anti-inflammatory (M2) to the inflammatory (M1) phenotype of macrophages. It also induces an inflammatory process by directly recruiting macrophages (acts as a chemoattractant) and by stimulating MCP1 and iNOS through the JNK-cJun-IFNγ-JAK2-STAT1-NOX4 pathway. Eventually, fetuin-A triggered inflammation leads to NAFLD, ATI, beta cell apoptosis, IR, and hence T2DM. Abbreviations: ATI, Adipose tissue inflammation; FFA, free fatty acid; IFN-γ, interferon-gamma; iNOS, inducible nitric oxide synthase; IR, insulin resistance; JAK 2, Janus kinase; MCP-1, monocyte chemoattractant protein-1; NAFLD, nonalcoholic liver disease; NOX4, NADPH oxidase 4; STAT1, signal transducer and activator of transcription; T2DM, type 2 diabetes mellitus; TLR-4, toll-like receptor-4.

Lipid-induced increment of fetuin-A exhibits inflammatory activity and triggers inflammation in different tissues, including adipocytes, leading to adipose tissue inflammation (ATI) and loss of insulin sensitivity. Fetuin-A has been observed to transport lipid throughout the body. A lipid-laden fetuin-A is considered a missing link between FFA–induced inflammation (Zhou et al., 2018). It promotes ATI by acting as an endogenous ligand for TLR-4, chemoattractant, and macrophage-polarizing agent (Figure 2). It has been found that fetuin-A exposes FFA to TLR-4 and triggers TLR-4-mediated expression of proinflammatory cytokines from adipocytes and macrophages using the TLR4-NFκB pathway (Tang et al., 2015). This was further proven by several *in vitro* and *in vivo* studies claiming that FFA-induced proinflammatory cytokine expression in adipocytes occurs only when both fetuin-A and TLR-4 are present, implying that fetuin-A acts as an endogenous ligand for TLR-4 in activating an inflammatory signaling pathway and resulting in ATI and IR (Pal et al., 2012).

Besides, macrophage migration and macrophage polarization from anti-inflammatory (M2) to pro-inflammatory phenotype (M1) are the other pivotal mechanisms through which FFA-fetuin-A complexes derive inflammation in adipose tissues (Chatterjee et al., 2013). According to a growing body of evidence, fetuin-A instigates inflammation by recruiting macrophages into inflamed adipose tissues and functioning as a chemoattractant, similar to monocyte chemoattractant protein-1 (MCP-1). It also induces ATI by transforming macrophages from M2 to M1, albeit the mechanism is still not well known. The recruited M1 macrophage then secretes proinflammatory cytokines that triggers inflammation and results in IR and T2DM (Pal et al., 2012; Chatterjee et al., 2013; Bhattacharya and Mukherjee, 2016). A more recent study by Chattopadhyay and his coworkers revealed that fetuin-A is a positive regulator of MCP-1 and inducible nitric oxide synthase (iNOS). This was demonstrated by considerable repression of MCP-1 and reduction of the macrophage content of adipose tissue from fetuin-A knockdown high fat diet-fed mice. In the fetuin-A knockdown mice, the expression of iNOS is significantly subdued and macrophage polarization flips from proinflammatory M1 to anti-inflammatory M2. This implies that fetuin-A upregulates the expression of both MCP-1 and iNOS that induce ATI through the JNK-cJun-IFNγ-JAK2-STAT1 pathway. Fetuin-A stimulates cJun N-terminal kinase (JNK) and cJun that increase the expression of interferon-gamma (IFN-γ) to stimulate the JAK2-STAT1-NOX4 cascades (Chattopadhyay et al., 2021).

Moreover, normal levels of intracellular fetuin-A have been found to play an antiapoptotic role by inhibiting the proteolytic cleavage and activation of caspases (Reynolds et al., 2005). However, excess fetuin-A secreted from pancreatic beta cells and/or circulating in the plasma, like in hyperlipidemic situations, elicits inflammation and damages to the beta cells (Mukhuty et al., 2017). Pancreatic β-cells treated with fetuin-A and FFA (palmitate) have been associated with beta cell failure and apoptosis, which is suggested to be mediated through the TLR4-signaling pathway (Shen et al., 2015). Consistently, a follow-up study by Mukhuty et al. (2022) unveiled that fetuin-A plays a critical role in triggering FFA-mediated beta cell inflammation, beta-cell dysfunction, and apoptosis by inducing lipotoxicity and activating the TLR4-NF-κB pathway in hyperlipidemic conditions. More interestingly, the apoptosis-inducing activity of fetuin-A has also been observed in cancer cell lines, such as mouse P388 leukemia and PC-3 prostate cancer models, with no effect on normal cell lines, suggesting its potential use as a selective anticancer agent. Fetuin-A has been proposed to induce apoptosis in cancer cells by raising the production of alkaline phosphatase (ALP), though the mechanism needs further elucidation (Yu and Tsai, 2001). Overall, fetuin-A plays a significant anti-apoptotic effect in normal circumstances, making it essential for cell survival. On the other hand, overexpression of fetuin-A induced by excess FFA in hyperlipidemic or other abnormal situations increases apoptotic activity by inducing inflammation in cells such as pancreatic beta cells, demonstrating its detrimental impact when produced excessively.

### Adipogenic role of fetuin-A

**FIGURE 3:**
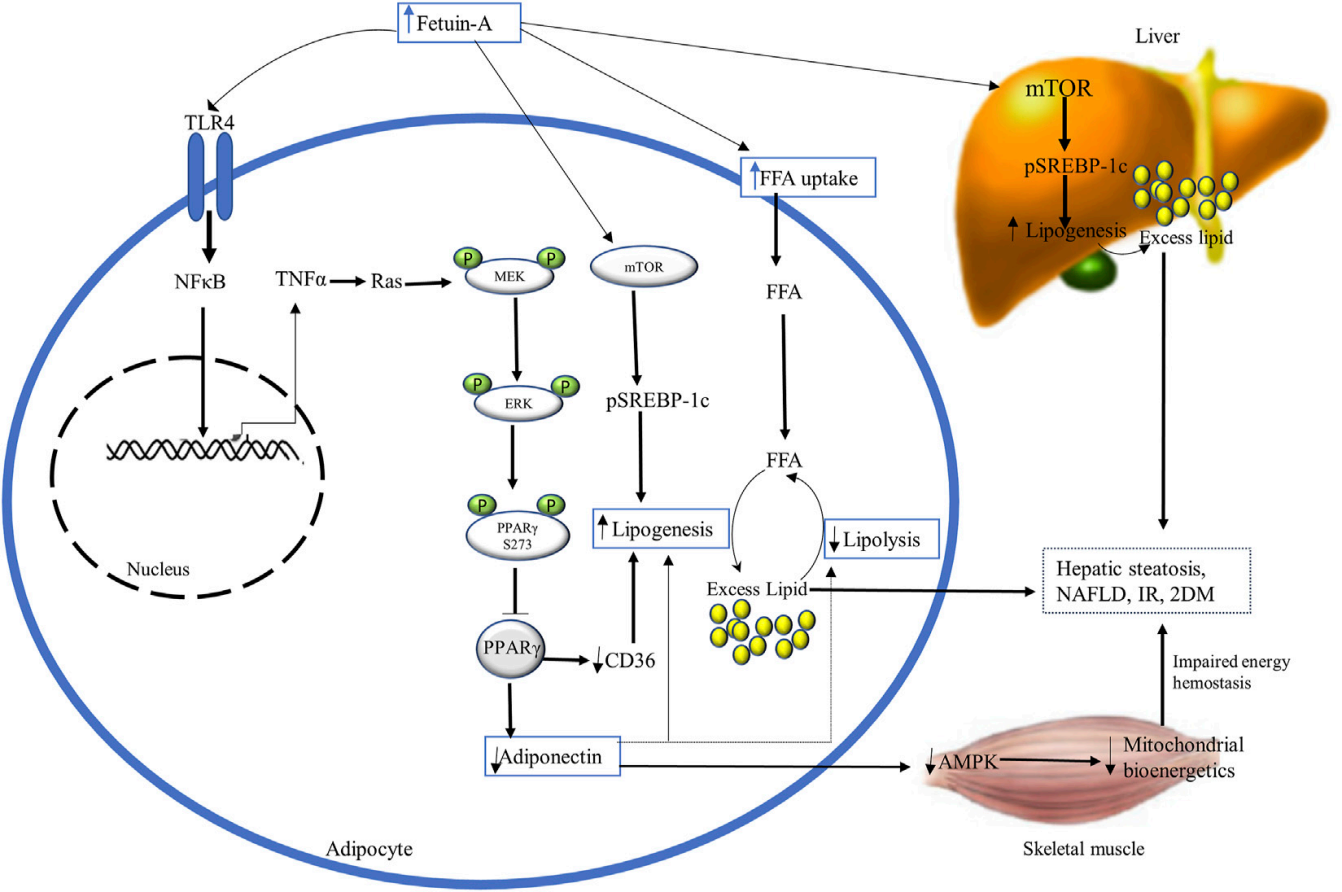
The diagrammatic illustration of the adipogenic role of fetuin-A. Excess fetuin-A release (from liver and adipocytes) may increase FFA uptake and accumulation in adipocytes and impair their function by inhibiting PPARγ and stimulating mTOR. Fetuin-A-mediated inhibition of PPARγ occurs through phosphorylation at serine 273 by TLR4-induced activation of NFκB-TNFα-Ras-MEK-ERK cascading. This suppression of PPARy lowers adiponectin levels and antagonizes its insulin-sensitizing and anti-inflammatory actions by impairing AMPK and mitochondrial bioenergetics. Besides, inhibition of PPARy downregulates the expression of CD36, resulting in lipogenesis and excess fat accumulations in adipocytes, disrupting insulin sensitivity. Excess fetuin-A also plays builds up excess fat in the liver and adipocytes by the stimulatory phosphorylation of mTOR that enhances the expression of SREBP-1c inducing lipogenesis. Ultimately, fetuin-A-induced disruption of mitochondrial bioenergetics and increased lipogenesis results in hepatic steatosis, NAFLD, IR, and T2DM. Abbreviations: AMPK, AMP-activated kinase; mTOR, phosphorylation of mammalian target of rapamycin; NFκB, nuclear factor κB; PPARy, Peroxisome proliferator-activated receptor-gamma; SREBP-1c, steroid regulatory element-binding protein-1c; TNF-α, tumor necrosis factor-alpha.

Fetuin-A has adipogenic properties that increase the uptake and storage of FFA in adipocytes, and impair adipocyte function by inhibitory phosphorylation of PPARγ and stimulatory phosphorylation of mammalian target of rapamycin (mTOR) (Figure 3) (Hennige et al., 2008; Dasgupta et al., 2010; Ix and Sharma, 2010). PPARγ is a nuclear transcription factor that serves as a master regulator of adipogenesis, lipid and glucose metabolism, and insulin sensitivity. It is essential to control the expression of several target genes, including adiponectin, fatty acid-binding proteins 4 (FABP4), and fatty acid translocase (FAT or CD36). Post-translational phosphorylation of PPARγ at Ser273 in adipose tissue dysregulates the expression of these target genes (Iwaki et al., 2003; Choi et al., 2010; Pal et al., 2012; Agarwal et al., 2017; Chattopadhyay et al., 2018).

Adiponectin is the main fat-derived athero-protective (antiatherogenic) and insulin-sensitizing (antidiabetic) adipokine that is involved in energy hemostasis and lipid mobilization (Arita et al., 1999; Haque et al., 2002). According to recent findings, fetuin-A has an inverse association and an inhibitory effect to adiponectin. It has been proposed that fetuin-A induces TLR-4 mediated antagonizing action on adiponectin *via* Wnt3a-PPARy signaling, inhibiting the insulin-sensitizing and anti-inflammatory effect of adiponectin (Sardana et al., 2021). Fetuin-A is reported to be an upstream regulator of PPARγ phosphorylation at serine 273 (S273) through the Ras-MEK-ERK pathway, which inhibits adiponectin activity (Das et al., 2021). It was proven that fetuin-A overexpression in high-fat diet-fed mice has shown a robust association with increased phosphorylation of PPARS273 in inflamed adipocytes, whereas fetuin-A knockdown inhibited it. The stimulatory effect of fetuin-A on MEK-ERK for PPARγS273 inhibitory phosphorylation is induced by TNFα-mediated Ras activation through TLR4-NFκB (Isayama et al., 2004). This ultimately results in decreased adiponectin expression that leads to disrupted AMP-activated protein kinase (AMPK) activation. Impaired AMPK negatively affects lipid mobilization and energy production in the mitochondria of skeletal muscle, losing insulin sensitivity and enhancing IR (Das et al., 2021).

Additionally, fetuin-A plays a central adipogenic role by cross-talking with FAT or CD36 through the inhibition of PPARy in adipocytes. CD36 is a key fatty acid-binding protein involved in regulating FFA uptake across the cell membrane in the heart and skeletal muscles. Experimental studies revealed that CD36 deficiency lowers FFA metabolism and uptake by myocytes, whereas its overproduction increases FFA metabolism and uptake (Bonen et al., 2004). CD36 expression is downregulated by fetuin-A-induced inhibitory phosphorylation of PPARy, resulting in an excess fat buildup that impairs insulin sensitivity (Hotamisligil et al., 1996; Bonen et al., 2004). According to observations from many animal studies, fetuin-A also increases the uptake and storage of FFA in adipocytes, resembling the activity of FABP-4. FABP4, also known as adipocyte protein 2 (aP2) or adipocyte FABP (A-FABP), is a major cytosolic adipocyte protein that regulates the intracellular mobilization of lipid (Hotamisligil et al., 1996). It is an important lipid chaperone that is involved in intracellular transport of FFA such as oleic and retinoic acid and also augments lipolysis in adipocytes. Both fetuin-A and FABP-4 reversibly bind, with high affinity, to hydrophobic ligands such as saturated and unsaturated long-chain fatty acids and other lipids (Cayatte et al., 1990; Kumbla et al., 1991; Furuhashi and Hotamisligil, 2008). It has been shown that FABP4 may link fatty acid metabolism to the expression of TNFα and possibly play a key role in the development of obesity and IR (Hotamisligil et al., 1996). In line with this, increased plasma FABP4 levels have been proven to have direct correlations with IR, metabolic syndrome, obesity, dyslipidemia, hypertension, and diabetes mellitus. This is accomplished through FABP4-dependent PPARγ inhibition that ubiquitinates and then degrades a key regulator of adipogenesis and insulin sensitivity, PPARγ. Which ultimately increases the accumulation of lipids in adipocytes (Furuhashi, 2019; Sun et al., 2020; Xiao et al., 2021). In the same way as FABP-4 does, fetuin-A accelerates the incorporation of exogenous fatty acids into cellular triglycerides including the liver and adipocytes through PPARγ phosphorylation. Fetuin-A knockout mice did not show weight gain when they were given a high-fat diet and showed lower serum levels of FFA and triglyceride (Mathews et al., 2002; Reinehr and Roth, 2008; Ix and Sharma, 2010). This revealed that fat accumulation in the liver, like in NAFLD, may be linked with the upregulation of fetuin-A levels (Sardana et al., 2021). Increased triglyceride accumulation and hepatic steatosis in NAFLD with elevated fetuin-A could be due to fetuin-A–induced phosphorylation of mTOR enhancing the expression of steroid regulatory element-binding protein-1c (SREBP-1c) (Mukhopadhyay et al., 2014). Altogether, fetuin-A and FABP4 have a synergistic action towards lipid deposition, and they have positive correlations with obesity, metabolic syndrome, and IR, but the relationship between them still needs further study (Kaess et al., 2012).

### The role of fetuin-A in insulin signaling

**FIGURE 4:**
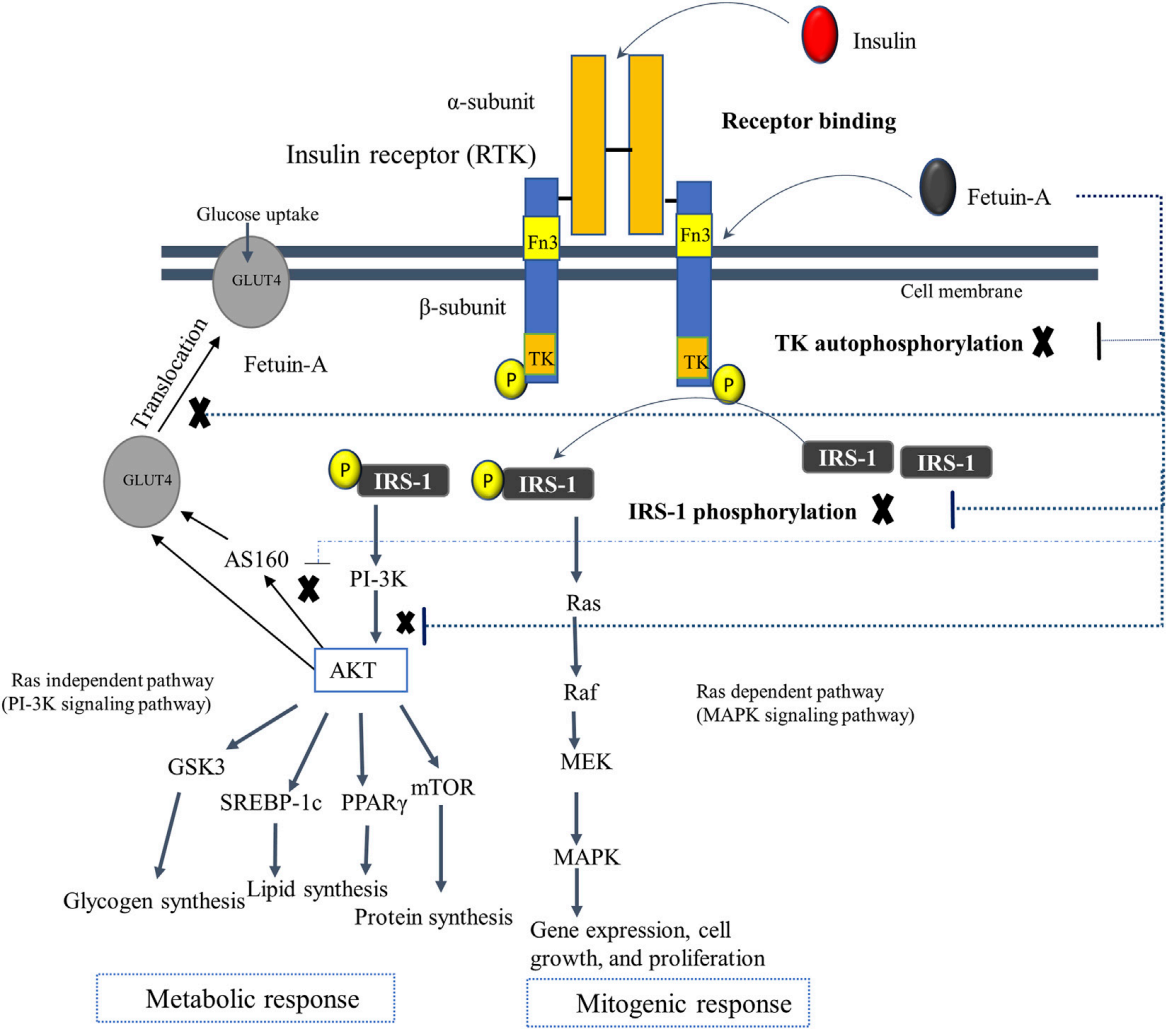
The postulated regulatory mechanism of fetuin-A in insulin signaling. Fetuin-A interacts with the tandem Fn3 situated in the extracellular domain of the transmembrane subunit of the RTK, which is away from the binding site of insulin. The D3 domain of fetuin-A is postulated to bind with the RTK. The interaction of fetuin-A with insulin receptor switches off the intracellular insulin signaling pathway by impairing the autophosphorylation of tyrosine kinase and IRS-1, as well as lowers glucose uptake by inhibiting phosphorylation of AKT, AS160, and GLUT4 (as shown in dotted lines), and thus controlling the overall insulin signaling pathway. Abbreviations: As160, Akt substrate of 160 kDa; Fn3, fibronectin type 3 domains; GLUT4, glucose transporter 4; GSK3, Glycogen synthase kinase 3; IRS-1, insulin receptor substrate-1; MAPK, Mitogen-activated protein kinase; MEK, MAPK kinase; PI-3k, Phosphoinositide 3-kinase; TK, Tyrosine kinase.

Numerous *in vivo* and *in vitro* studies have revealed that fetuin-A regulates insulin receptor sensitivity and insulin signaling pathway at the insulin receptor level (Stefan et al., 2008; Wojtysiak-Duma et al., 2010; Song et al., 2011). Fetuin-A acts as a natural inhibitor of the insulin receptor known as the receptor tyrosine kinase (RTK). The RTK, which is found abundantly in peripheral tissues such as liver, muscle, and adipose tissues, comprises two ligand (insulin) binding alpha subunits and two intracellular insulin signals mediating beta subunits. Fetuin-A is identified to bind with RTK away from the binding site of insulin, though it has not yet been well known which domain of fetuin-A interacts with RTK. Indeed, there are some reports unveiling the increased likelihood and high affinity of the D3 domain of fetuin-A for binding directly and non-competitively to the insulin receptor, but there needs to be more research to verify this finding (Brown et al., 1992; Brown et al., 1992). In particular, fetuin-A attaches to the tandem fibronectin type 3 (fn3) domains situated in the extracellular domain of the transmembrane subunit of RTK. This interaction of fetuin-A with the RTK turns off the intracellular insulin signaling pathway by impeding the autophosphorylation of tyrosine kinase and insulin receptor substrate-1 (IRS-1). Besides, fetuin-A may decrease glucose uptake by inhibiting the phosphorylation of AKT, AS160, and GLUT4, and thus control the insulin signaling pathway (Figure 4) (Stefan et al., 2008; Wojtysiak-Duma et al., 2010). This is congruent with other mouse studies indicating that fetuin-A null mice have been observed to have enhanced basal and insulin-mediated phosphorylation of RTK, increased glucose clearance, and improved insulin sensitivity (Stefan et al., 2006b; Reinehr and Roth, 2008). Another study has also found that fetuin-A levels are positively correlated with fasting and elevated insulin levels, and negatively associated with insulin sensitivity (Stefan et al., 2006b).

Elevated circulating levels of fetuin-A is, however, linked with impaired insulin sensitivity that leads to the development of IR and related comorbidities such as hypertriglyceridemia, obesity, impaired glucose tolerance, T2DM, metabolic syndrome, NAFLD, and early-stage CKD (Ix and Sharma, 2010; Song et al., 2011). Fetuin-A mediated development of IR has been documented in a number of previous human studies demonstrating the positive association between fetuin-A level and IR (Weikert et al., 2008; Erdmann et al., 2012). This is corroborated by mice studies that show the development of IR in wild-type mice on exogenous fetuin-A therapy and increment of insulin sensitivity in fetuin-A deficient mice (Mathews et al., 2002; Hennige et al., 2008). Fetuin-A triggered development of IR and associated comorbidities are mediated by several interwoven molecular mechanisms. One is that fetuin-A mediates inhibition of RTK and disrupts the insulin signaling pathway, leading to IR. In addition, fetuin-A attenuates insulin sensitivity and is involved in the occurrence of IR by inducing lipid deposition in the adipocytes and liver that causes inflammation (Pal et al., 2012; Trepanowski et al., 2015; Gerst et al., 2017). It also enhances lipid-induced IR through the interactions of FFA with TLR-4 that activate transcription factor NF-κB and activator protein-1 (AP-1) or Fos/Jun. This induces the production of inflammatory cytokines that further intensifies ATI and increases IR (Pal et al., 2012). Moreover, fetuin-A antagonizes the insulin-sensitizing properties of adiponectin, which worsen beta cell inflammation and IR (Chatterjee et al., 2013).

Fetuin-A also aggravates IR by inducing fetuin-A secretion from pancreatic beta cells, which promotes macrophages migration and transformation into the M1 phenotype in islets, elicits beta cell inflammation, and impairs their function. (Agarwal et al., 2017; Mukhuty et al., 2017; Mukhuty et al., 2021a; Mukhuty et al., 2022). This fetuin-A-mediated chronic beta cell inflammation occurs in hyperlipidemic conditions and is linked with beta cell apoptosis, mass reduction, and dysfunction, leading to decreased insulin secretion and worsening of IR (Hennige et al., 2008; Shen et al., 2015). A recent publication deciphered that a fatty liver-derived fetuin-A may impair glucose-induced insulin secretion and plays a diabetogenic role. It has been found to mediate the metabolic crosstalk between the fatty liver and the fatty pancreas, which augments local pancreatic inflammation and beta cell failure. This lowers glucose-induced insulin secretion and speeds up the progression to overt T2DM (Gerst et al., 2017). Fetuin-A triggers beta cell inflammation (and hampers insulin secretion) *via* its direct proinflammatory, TLR4-independent, or TLR4-dependent effects (Gerst et al., 2017). Lipid-induced fetuin-A secretion from beta cells and/or fatty liver directly instigates beta cell inflammation and apoptosis and reduced insulin secretion (Hennige et al., 2008; Shen et al., 2015). Fetuin-A also inhibits glucose-induced insulin secretion by activating JNK in a Ca^2+^-dependent but TLR4-independent manner (Gerst et al., 2017). Activation of JNK in pancreatic beta cells has also been observed to be linked with glucose intolerance and IR in previous investigations (Kim et al., 2008; Lanuza-Masdeu et al., 2013).

Notably, fetuin-A, in the presence of FFA, is a potent activator of pancreatic adipocytes and pre-adipocytes to produce chemokine (MCP-1 and IL-8) and cytokine (IL-6) in TLR4 dependent manner. Fetuin-A has also been demonstrated to stimulate islet resident macrophages expressing cytokines (IL-6 and IL-1β) through the TLR4-NK-κB pathway. The released chemo attractants increase macrophage or monocytes infiltration of islets that express cytotoxic cytokines triggering a local proinflammatory response in the pancreatic adipocytes and islet macrophages (Gerst et al., 2017). Consequently, fetuin-A and FFA induced chronic beta cell inflammation that results in beta cell death and mass reduction hampers insulin secretion (Ehses et al., 2007; Richardson et al., 2009; Eguchi et al., 2012; Nackiewicz et al., 2014). Consistently, another study indicates that the combination of FFA and fetuin-A stimulates TLR4-NF-κB induced inflammation in pancreatic beta cells and impairs insulin secretion (Mukhuty et al., 2021b). In hyperlipidemic circumstances, a significant reduction of insulin gene expression and secretion is owing to the downregulation of Pdx1, GLUT2, and pAkt, as well as the upregulation of FoxO1. Concomitantly, there is an increment of islet-derived fetuin-A expression hyperlipidemic conditions, which further reduces glucose-induced insulin secretion from beta cells. It has been confirmed that inhibiting fetuin-A, TLR4, and NF-κB in mouse models prevents the combinatorial negative impacts of FFA and fetuin-A on pancreatic β-cells while eliciting insulin secretion. While silencing of TLR4 in islets averts the action of FFA and fetuin-A, inhibition of NF-κB reduces fetuin-A secretion and restores insulin secretion (Mukhuty et al., 2021b).

Furthermore, fetuin-A may impair insulin sensitization and results in IR by increasing the plasma levels of blood glucose. Increased fetuin gene expression in response to higher blood glucose levels reveals a positive correlation between plasma fetuin-A and glucose levels. Fetuin-A downregulates the glucose uptake of skeletal muscle by suppressing phosphorylation of Akt, AS160, and intracellular GLUT4 translocation to the plasma membrane, resulting in obesity, IR, and NAFLD (Ou et al., 2012). This can be corroborated by fetuin-A mediated interruption of energy homeostasis in the adipocytes of insulin-resistant obese mice by disrupting energy sensors, NAD^+^ dependent deacetylase sirtuin1 (SIRT1), and AMPK (Gerst et al., 2017). Overall, elevated levels of fetuin-A potentially cause impaired glycemic control, IR, and overt T2DM ascribed to fetuin-A mediated blockade of insulin signaling, TLR-4 activation, macrophage migration, and polarization, adipocyte dysfunction, liver lipid deposition, inflammation, and fibrosis, as well as FFA-mediated inflammation of pancreatic beta cells.

### Atherogenic role of fetuin-A

More intriguingly, due to its underlying causative link with IR and adipocyte dysfunction, fetuin-A has atherogenic features that worsen atherosclerosis and CVD (Heiss et al., 1991). Serum fetuin-A levels were found to be positively correlated with carotid arterial stiffness that measures atherosclerotic changes, speculating fetuin-A as an atherogenic factor rather than as a vascular calcification inhibitor, mainly during the early stages of atherosclerosis (Mori et al., 2006). The severity and hard plaque formation of atherosclerosis increase as fetuin-A levels decrease due to the lowering of its role as a vascular calcification inhibitor. When its levels rise, however, fetuin-A may have an atherogenic effect, showing its dual role in the formation of atherosclerosis. Similarly, both elevated and reduced fetuin-A may be linked with increased cardiovascular events. CVD may be caused by vascular calcification associated with low fetuin-A, as well as IR and dyslipidemia linked to elevated fetuin-A levels, revealing the biphasic roles of fetuin-A in CVD etiology (Mori et al., 2012). Taken together, while high fetuin-A exacerbates the early stages of CVD due to its enhancing effects on IR and dyslipidemia, it has protective effects in the later stages of CVD by abrogating ectopic vascular calcification (Zhao et al., 2013; Gerdes et al., 2014).

### The role of fetuin-A in regulating protease enzymes

Fetuin-A also exhibits molecular functions as an enzyme regulator, modulating a wide range of proteases. It has been demonstrated to interact with proteases in both positive and negative ways, such as metalloproteinase, papain, calpain, cathepsin, caspases, and matriptase-2 (Jahnen-Dechent et al., 2011). Fetuin-A has been discovered to be an endogenous inhibitor of metalloprotease activities, including matrix metalloprotease-3, matrix metalloprotease-9, and meprin metalloprotease (meprin α and β) (Tajirian et al., 2000; Leite-Browning et al., 2002; Hedrich et al., 2010). Likewise, it negatively regulates lysosomal cysteine proteases, such as m-calpain, cathepsins L, and cathepsin V (Nakamura et al., 1999; Tajirian et al., 2000; Mellgren and Huang, 2007; Broder and Becker-Pauly, 2013). The interactions of fetuin-A and these proteases are hypothesized to have a role in the regulation of tumorigenesis and tumor progression (Leite-Browning et al., 2002; Leite-Browning et al., 2004; Kundranda et al., 2005). Kundranda et al. (2005) found that fetuin-A promotes the tumorigenesis of Lewis lung carcinoma *via* adhesive-dependent and adhesive-independent pathways. In addition, bovine fetuin-A was demonstrated to bind to the plasma membrane of squamous and spindle cell carcinoma cells (Leite-Browning et al., 2002). Consistently, another study suggests that fetuin-A contributes to the early stages of skin tumorigenesis (Leite-Browning et al., 2004).

Previous studies indicated that fetuin-A is the inhibitor of caspases that are involved in apoptotic cascades. Intracellular fetuin-A directly interacts with caspase 3, 8, and 9 and prevents them from being cleaved and activated, indicating its antiapoptotic roles (Reynolds et al., 2005). Generally, the anti-proteolytic activity of fetuin-A against different proteases, including caspases, is localized in its D1 and D2 domains (Ray et al., 2003). Furthermore, fetuin-A has been observed to interact with a member of type II transmembrane serine proteases, known as matriptase-2 (Stirnberg et al., 2015). More recently, fetuin-A is considered a putative substrate of matriptase-2, in addition to hemojuvelin and matriptase-2 itself (Silvestri et al., 2008; Stirnberg et al., 2015). An *in vitro* study by Stirnberg et al. (2015) identified matriptase-2 utilizing fetuin-A as a substrate and suppresses its activity. This is done through proteolytic processing of the Arg and Lys residues of the CP of fetuin-A to yield a disulfide-linked two-chain form. The expression of hepcidin, which is a liver-derived key regulator of iron homeostasis, was suggested to be inducted by fetuin-A. This provides new insights into the implication of fetuin-A in iron homeostasis (Stirnberg et al., 2015).

### Other biological functions of fetuin-A

Fetuin-A has also been postulated to play an important physiological role in brain physiology, such as brain development and/or protection (Elsas et al., 2013). This is evident from the abundance of fetuin-A in the CSF and serum throughout brain development during intrauterine life, its anti-inflammatory and probable neuroprotective function in ischemic stroke, and its interactions with growth factors or cytokines (Wang et al., 2010; Elsas et al., 2013; Harris et al., 2013). More interestingly, fetuin-A has been shown to possess opsonic properties that enhance endocytosis and modulate innate immunity (Li et al., 2011). For several decades, fetuin-A has been known to be an opsonizing serum protein with a strong affinity not only for mineral complexes but also for debris. It influences dendritic cell phagocytosis of microparticles, macrophage phagocytosis of apoptotic cells, and opsonization of phospholipid particles (Van Oss et al., 1974; Lewis and Andre, 1980; Lewis and Andre, 1981; Moghimi and Hunter, 2001; Jersmann et al., 2003; Thiele et al., 2003).

## Discussion

It was 80 years ago that fetuin-A was first discovered in the blood of a fetal calf, and later, 60 years ago, it was first isolated in human blood. Nowadays, the molecular structure, biosynthesis, and several biological functions of fetuin-A have been illustrated. Hence, this article comprehensively reviews several recent publications that are useful in providing current and up-to-date information regarding fetuin-A, which will shed light on easing the understanding of this complex topic for readers. The molecular structure of fetuin-A has been extensively studied in different species, both in intrauterine and extrauterine life. According to the coding sequences of 16 primate species, fetuin-A has shown molecular evolution. Its D3 domain is remarkably characterized by accelerated sequence evolution, which is driven by positive Darwinian selection, but by not GC-biased gene conversion. Besides, the D3 domain is associated with a significant accumulation of proteolytic cleavage sites, likely due to the evolution of differential cleavage site patterns across primates. But this area remains complex and needs further research to demonstrate its structure as well as to show the structural differences among different species.

Regarding biosynthesis, fetuin-A is produced primarily in the liver and, to a lesser extent, in other tissues. Its synthesis starts with the expression of the AHSG gene located on the 3q27 human chromosome locus. The process of fetuin-A expression is found to be highly regulated, although it is still vague and warrants further elucidation. A plethora of data has also documented that human fetuin-A undergoes PTMs, such as glycosylation, proteolysis, folding, and phosphorylation to form mature fetuin-A, although it is still less clearly understood and variable.

Fetuin-A is identified to have diverse physiological functions in human beings. In normal circumstances, it is involved in various regulatory roles, including regulation of insulin signaling pathway, calcium metabolism, and enzyme activities. Furthermore, collective evidence confirmed that fetuin may play an important role in embryonic development, as well as other functions in the human body, such as antiapoptotic activities, brain development, endocytosis, opsonizing activities, and transport function. This indicates that fetuin is a very essential protein in the maintenance of normal body functioning. However, when the level of fetuin-A is out of the normal range (either increased, decreased, or even absent) due to several underlying causes, it is demonstrated to have a pathological role that is associated with the development of a broad range of clinical disorders. Abnormal levels of fetuin-A or fetuin-A in abnormal conditions, such as hyperlipidemic state, contribute to a major role in the development of various diseases, either directly or indirectly. Given its critical involvement in a vast array of disorders, fetuin-A may be used as a potential diagnostic marker and therapeutic target for different pathologies in the future.

A number of biological functions (whether physiological or pathological roles) ascribed to fetuin-A has increased exponentially in the last few decades. However, despite the multifaceted functions of fetuin-A, further thorough studies need to be done to fully understand all its possible roles in humans. In spite of our extensive effort to provide comprehensive and up-to-date reviews regarding the structure, biosynthesis, and biological roles of fetuin-A, this review gave more emphasis on the physiological roles of fetuin-A. Besides, although it has been found to be a major player in the development of several disorders, and it may have therapeutic and diagnostic roles, this work did not incorporate the details on these areas of fetuin-A.

## Concluding remarks

In summary, fetuin-A is a multidomain hepato-adipokine protein that is primarily produced from the liver and encoded by the AHSG gene located on the 3q27 human chromosome locus. It is a pleiotropic protein that plays a pivotal role in a wide range of physiological and pathological activities. Fetuin-A is involved in local calcium mineral scavenging, insulin signaling, modulating macrophage polarization, brain development, endocytosis, and cysteine protease inhibitory activities. Besides, it exhibits functions in different pathologies such as IR, T2DM, metabolic syndrome, sepsis, brain lesions, NAFLD, atherosclerosis, CVD, and autoimmune disorders. These diverse effects of fetuin-A are brought about by its interaction with a plethora of receptors, like insulin, growth factors, TGF-β, and TLR receptors. Taken together, fetuin-A can serve as a biomarker and a target for the diagnosis and treatment of clinical diseases associated with it.

## Abbreviations

AHSG, α2-Heremans-Schmid glycoprotein; APP, acute phase protein; BMP, bone morphogenetic protein; C/EBP)-β, CCAAT enhancer-binding proteins-β; CKD, chronic kidney disease; CRP, C reactive protein; CVD, cardiovascular disease; EGF, epidermal growth factor; ESRD, End stage renal disease; FAM20C, Family with sequence similarity 20 member C; HMGBP1, high mobility group box protein 1; IBD, inflammatory bowel disease; IFN-γ, interferon-γ; IL-1, interleukin-1; IR, insulin resistance; IRS-1, insulin receptor substrate-1; mTOR, mammalian target of rapamycin; NAFLD, Non-alcoholic fatty liver disease; NF-1, nuclear factor-1; PAMP, pathogen-associated molecular pattern; PASI, RTK, receptor tyrosine kinase; SREBP-1c, steroid regulatory element–binding protein-1c; SS, signal sequence; TGF-β, transforming growth factor-β; TGF, transforming growth factor; TLR-4, toll-like receptor 4; TNF-α, tumor necrosis factor-alpha.

## References

[B1] Abebe E. C. Muche Z. T. Behaile T/Mariam A. Mengie Ayele T. Mekonnen Agidew M. Teshome Azezew M. (2022). Role of fetuin-A in the pathogenesis of psoriasis and its potential clinical applications. Clin. Cosmet. Investig. Dermatol. 15, 595–607. 10.2147/CCID.S356801 PMC9005232 35422648

[B2] Afrisham R. Sadegh-Nejadi S. Meshkani R. Emamgholipour S. Paknejad M. (2020). Effect of circulating exosomes derived from normal-weight and obese women on gluconeogenesis, glycogenesis, lipogenesis and secretion of FGF21 and fetuin A in HepG2 cells. Diabetol. Metab. Syndr. 12 (1), 32. 10.1186/s13098-020-00540-4 32322309 PMC7161281

[B3] Agarwal S. Chattopadhyay M. Mukherjee S. Dasgupta S. Mukhopadhyay S. Bhattacharya S. (2017). Fetuin-A downregulates adiponectin through Wnt-PPARγ pathway in lipid induced inflamed adipocyte. Biochim. Biophys. Acta. Mol. Basis Dis. 1863 (1), 174–181. 10.1016/j.bbadis.2016.10.002 27720679

[B4] An W. S. Lee S. M. Son Y. K. Kim S. E. Kim K. H. Han J. Y. (2012). Omega-3 fatty acid supplementation increases 1, 25-dihydroxyvitamin D and fetuin-A levels in dialysis patients. Nutr. Res. 32 (7), 495–502. 10.1016/j.nutres.2012.06.005 22901557

[B5] Andersen G. Burgdorf K. S. Sparsø T. Borch-Johnsen K. Jorgensen T. Hansen T. (2008). AHSG tag single nucleotide polymorphisms associate with type 2 diabetes and dyslipidemia: Studies of metabolic traits in 7, 683 white Danish subjects. Diabetes 57 (5), 1427–1432. 10.2337/db07-0558 18316360

[B6] Arita Y. Kihara S. Ouchi N. TakahashiM. Maeda K. Miyagawa J. (1999). Paradoxical decrease of an adipose-specific protein, adiponectin, in obesity. Biochem. Biophys. Res. Commun. 257 (1), 79–83. 10.1006/bbrc.1999.0255 10092513

[B7] Banine F. Gangneux C. Mercier L. Le CAm A. Salier J. P. (2000). Positive and negative elements modulate the promoter of the human liver-specific alpha2-HS-glycoprotein gene. Eur. J. Biochem. 267 (4), 1214–1222. 10.1046/j.1432-1327.2000.01119.x 10672033

[B8] Bhattacharya S. Mukherjee S. (2016). Lipid links inflammation, immunity and insulin resistance to cause epidemic diabetes. Curr. Sci. 110, 1922. 10.18520/cs/v110/i10/1922-1928

[B9] Binkert C. Demetriou M. Sukhu B. SzwerasM. Tenenbaum H. C. Dennis J. W. (1999). Regulation of osteogenesis by fetuin. J. Biol. Chem. 274 (40), 28514–28520. 10.1074/jbc.274.40.28514 10497215

[B10] Bonen A. Campbell S. E. Benton C. R. Chabowski A. Coort S. L. M. Han X. X. (2004). Regulation of fatty acid transport by fatty acid translocase/CD36. Proc. Nutr. Soc. 63 (2), 245–249. 10.1079/PNS2004331 15294038

[B11] Borsky P. Fiala Z. Andrys C. (2021). C-reactive protein, chemerin, fetuin-A and osteopontin as predictors of cardiovascular risks in persons with psoriasis vulgaris. Czec Republic: Physiological Research. 10.33549/physiolres.934654 PMC8820567 33982577

[B12] Broder C. Becker-Pauly C. (2013). The metalloproteases meprin α and meprin β: Unique enzymes in inflammation, neurodegeneration, cancer and fibrosis. Biochem. J. 450 (2), 253–264. 10.1042/BJ20121751 23410038 PMC3573791

[B13] Brown W. Dziegielewska K. (1997). Friends and relations of the cystatin superfamily—New members and their evolution. Protein Sci. 6 (1), 5–12. 10.1002/pro.5560060102 9007972 PMC2143511

[B14] Brown W. Dziegielewska K. Saunders N. (1992). Fetuin‐an old friend revisited. Bioessays 14 (11), 749–755. 10.1002/bies.950141105 1285422

[B15] Brown W. M. Dziegielewska K. M. Saunders N. R. Christie D. L. Nawratil P. Muller-Esterl W. (1992). The nucleotide and deduced amino acid structures of sheep and pig fetuin: Common structural features of the mammalian fetuin family. Eur. J. Biochem. 205 (1), 321–331. 10.1111/j.1432-1033.1992.tb16783.x 1372866

[B16] Caglar K. Yilmaz M. I. Saglam M. Cakir E. Acikel C. Eyileten T. (2008). Short-term treatment with sevelamer increases serum fetuin-a concentration and improves endothelial dysfunction in chronic kidney disease stage 4 patients. Clin. J. Am. Soc. Nephrol. 3 (1), 61–68. 10.2215/CJN.02810707 18057307 PMC2390988

[B17] Cayatte A. Kumbla L. Subbiah M. (1990). Marked acceleration of exogenous fatty acid incorporation into cellular triglycerides by fetuin. J. Biol. Chem. 265 (10), 5883–5888. 10.1016/s0021-9258(19)39445-1 1690716

[B18] Cerdà‐Costa N. Xavier Gomis‐Rüth F. (2014). Architecture and function of metallopeptidase catalytic domains. Protein Sci. 23 (2), 123–144. 10.1002/pro.2400 24596965 PMC3926739

[B19] Chatterjee P. Seal S. Mukherjee S. Kundu R. Ray S. Mukhopadhyay S. (2013). Adipocyte fetuin-A contributes to macrophage migration into adipose tissue and polarization of macrophages. J. Biol. Chem. 288 (39), 28324–28330. 10.1074/jbc.C113.495473 23943623 PMC3784748

[B20] Chattopadhyay D. Das S. Guria S. Basu S. Mukherjee S. (2021). Fetuin-A regulates adipose tissue macrophage content and activation in insulin resistant mice through MCP-1 and iNOS: Involvement of IFNγ-JAK2-STAT1 pathway. Biochem. J. 478 (22), 4027–4043. 10.1042/BCJ20210442 34724561

[B21] Chattopadhyay M. Mukherjee S. Chatterjee S. K. Chattopadhyay D. Das S. Majumdar S. S. (2018). Impairment of energy sensors, SIRT1 and AMPK, in lipid induced inflamed adipocyte is regulated by Fetuin A. Cell. Signal. 42, 67–76. 10.1016/j.cellsig.2017.10.005 29030114

[B22] Chen H-Y. Chiu Y-L. Hsu S-P. Pai M. F. Lai C. F. Peng Y. S. (2009). Association of serum fetuin A with truncal obesity and dyslipidemia in non-diabetic hemodialysis patients. Eur. J. Endocrinol. 160 (5), 777–783. 10.1530/EJE-08-0813 19228823

[B23] Choi J. H. Banks A. S. Estall J. L. Kajimura S. Bostrom P. Laznik D. (2010). Anti-diabetic drugs inhibit obesity-linked phosphorylation of PPARgamma by Cdk5. Nature 466 (7305), 451–456. 10.1038/nature09291 20651683 PMC2987584

[B24] Choi K. M. Han K. A. Ahn H. J. Lee S. Y. Hwang S. Y. Kim B. H. (2013). The effects of caloric restriction on F etuin‐A and cardiovascular risk factors in rats and humans: A randomized controlled trial. Clin. Endocrinol. 79 (3), 356–363. 10.1111/cen.12076 23067229

[B25] Cuppari A. Körschgen H. Fahrenkamp D. Schmitz C. Guevara T. Karmilin K. (2019). Structure of mammalian plasma fetuin-B and its mechanism of selective metallopeptidase inhibition. IUCrJ 6 (2), 317–330. 10.1107/S2052252519001568 PMC6400186 30867929

[B26] Dabrowska A. M. Tarach J. S. Wojtysiak-Duma B. Duma D. (2015). Fetuin-A (AHSG) and its usefulness in clinical practice. Review of the literature. Biomed. Pap. Med. Fac. Univ. Palacky. Olomouc Czech. Repub. 159 (3), 352–359. 10.5507/bp.2015.018 25916279

[B27] Das S. Chattopadhyay D. Chatterjee S. K. Mondal S. A. Majumdar S. S. Mukhopadhyay S. (2021). Increase in PPARγ inhibitory phosphorylation by fetuin—a through the activation of ras-MEK-ERK pathway causes insulin resistance. Biochim. Biophys. Acta. Mol. Basis Dis. 1867 (4), 166050. 10.1016/j.bbadis.2020.166050 33359696

[B28] Dasgupta S. Bhattacharya S. Biswas A. Majumdar S. S. Mukhopadhyay S. Ray S. (2010). NF-kappaB mediates lipid-induced fetuin-A expression in hepatocytes that impairs adipocyte function effecting insulin resistance. Biochem. J. 429 (3), 451–462. 10.1042/BJ20100330 20482516

[B29] Demetriou M. Binkert C. Sukhu B. Tenenbaum H. C. Dennis J. W. (1996). Fetuin/alpha2-HS glycoprotein is a transforming growth factor-beta type II receptor mimic and cytokine antagonist. J. Biol. Chem. 271 (22), 12755–12761. 10.1074/jbc.271.22.12755 8662721

[B30] Denecke B. Gräber S. Schäfer C. Heiss A. Woltje M. Jahnen-Dechent W. (2003). Tissue distribution and activity testing suggest a similar but not identical function of fetuin-B and fetuin-A. Biochem. J. 376 (1), 135–145. 10.1042/BJ20030676 12943536 PMC1223762

[B31] Dietzel E. Wessling J. Floehr J. Schafer C. Ensslen S. Denecke B. (2013). Fetuin-B, a liver-derived plasma protein is essential for fertilization. Dev. Cell. 25 (1), 106–112. 10.1016/j.devcel.2013.03.001 23562279

[B32] Dziegielewska K. Brown W. (1995). Molecular Biology intelligence unit: Fetuin. Austin, TX: RG Landes Co, 59ą60.

[B33] Eguchi K. Manabe I. Oishi-Tanaka Y. Ohsugi M. Kono N. Ogata F. (2012). Saturated fatty acid and TLR signaling link β cell dysfunction and islet inflammation. Cell. Metab. 15 (4), 518–533. 10.1016/j.cmet.2012.01.023 22465073

[B34] Ehses J. A. Perren A. Eppler E. Ribaux P. Pospisilik J. A. Maor-Cahn R. (2007). Increased number of islet-associated macrophages in type 2 diabetes. Diabetes 56 (9), 2356–2370. 10.2337/db06-1650 17579207

[B35] Elsas J. Sellhaus B. Herrmann M. Kinkeldey A. Weis J. Jahnen-Dechent W. (2013). Fetuin‐A in the developing brain. Dev. Neurobiol. 73 (5), 354–369. 10.1002/dneu.22064 23109215

[B36] Erdmann J. Salmhofer H. Knauß A. Mayr M. Wagenpfeil S. Sypchenko O. (2012). Relationship of fetuin-A levels to weight-dependent insulin resistance and type 2 diabetes mellitus. Regul. Pept. 178 (1-3), 6–10. 10.1016/j.regpep.2012.02.004 22387701

[B37] Falquerho L. Paquereau L. Vilarem M. J. GalaS S. Patey G. Le CAm A. (1992). Functional characterization of the promoter of pp63, a gene encoding a natural inhibitor of the insulin receptor tyrosine kinase. Nucleic Acids Res. 20 (8), 1983–1990. 10.1093/nar/20.8.1983 1579501 PMC312316

[B38] Floehr J. Dietzel E. Schmitz C. (2016). Down-regulation of the liver-derived plasma protein fetuin-B mediates reversible female infertility. MHR Basic Sci. reproductive Med., 1–11. 10.1093/molehr/gaw068 27733488

[B39] Furuhashi M. (2019). Fatty acid-binding protein 4 in cardiovascular and metabolic diseases. J. Atheroscler. Thromb. 26, 216–232. 10.5551/jat.48710 30726793 PMC6402888

[B40] Furuhashi M. Hotamisligil G. S. (2008). Fatty acid-binding proteins: Role in metabolic diseases and potential as drug targets. Nat. Rev. Drug Discov. 7 (6), 489–503. 10.1038/nrd2589 18511927 PMC2821027

[B41] Gejyo F. Chang J. Bürgi W. Schmid K. Offner G. D. Troxler R. F. (1983). Characterization of the B-chain of human plasma alpha 2HS-glycoprotein. The complete amino acid sequence and primary structure of its heteroglycan. J. Biol. Chem. 258 (8), 4966–4971. 10.1016/s0021-9258(18)32522-5 6833285

[B42] Gerdes S. Osadtschy S. Buhles N. Baurecht H. Mrowietz U. (2014). Cardiovascular biomarkers in patients with psoriasis. Exp. Dermatol. 23 (5), 322–325. 10.1111/exd.12381 24660963

[B43] Gerst F. Wagner R. Kaiser G. Panse M. Heni M. Machann J. (2017). Metabolic crosstalk between fatty pancreas and fatty liver: Effects on local inflammation and insulin secretion. Diabetologia 60 (11), 2240–2251. 10.1007/s00125-017-4385-1 28791439

[B44] Ghaffari A. Rafraf M. Navekar R. (2017). Effects of turmeric on homocysteine and fetuin-A in patients with nonalcoholic fatty liver disease: A randomized double-blind placebo-controlled study. 10.3746/pnf.2022.27.1.37 PMC9007706 35465117

[B45] Haglund Å. C. Ek B. Ek P. (2001). Phosphorylation of human plasma alpha2-Heremans-Schmid glycoprotein (human fetuin) *in vivo*. Biochem. J. 357 (2), 437–445. 10.1042/0264-6021:3570437 11439093 PMC1221970

[B46] Haque W. Shimomura I. Matsuzawa Y. Garg A. (2002). Serum adiponectin and leptin levels in patients with lipodystrophies. J. Clin. Endocrinol. Metab. 87, 2395. 10.1210/jcem.87.5.8624 11994394

[B47] Harris V. K. Donelan N. Yan Q. J. Clark K. Touray A. Rammal M. (2013). Cerebrospinal fluid fetuin-A is a biomarker of active multiple sclerosis. Mult. Scler. 19 (11), 1462–1472. 10.1177/1352458513477923 23439582

[B48] Häusler M. Schäfer C. Osterwinter C. Jahnen-Dechent W. (2009). The physiologic development of fetuin-a serum concentrations in children. Pediatr. Res. 66 (6), 660–664. 10.1203/PDR.0b013e3181bc3f60 19690510

[B49] Hedrich J. Lottaz D. Meyer K. Yiallouros I. Jahnen-Dechent W. Stocker W. (2010). Fetuin-A and cystatin C are endogenous inhibitors of human meprin metalloproteases. Biochemistry 49 (39), 8599–8607. 10.1021/bi1004238 20806899

[B50] Heiss A. DuChesne A. Denecke B. Grotzinger J. Yamamoto K. Renne T. (2003). Structural basis of calcification inhibition by α2-HS glycoprotein/fetuin-A: Formation of colloidal calciprotein particles. J. Biol. Chem. 278 (15), 13333–13341. 10.1074/jbc.M210868200 12556469

[B51] Heiss G. Sharrett A. R. Barnes R. Chambless L. E. SzkloM. Alzola C. (1991). Carotid atherosclerosis measured by B-mode ultrasound in populations: Associations with cardiovascular risk factors in the ARIC study. Am. J. Epidemiol. 134 (3), 250–256. 10.1093/oxfordjournals.aje.a116078 1877584

[B52] Hennige A. M. Staiger H. Wicke C. Machicao F. Fritsche A. Haring H. U. (2008). Fetuin-A induces cytokine expression and suppresses adiponectin production. PloS one 3 (3), e1765. 10.1371/journal.pone.0001765 18335040 PMC2258416

[B53] Hotamisligil G. S. Johnson R. S. Distel R. J. Ellis R. Papaioannou V. E. Spiegelman B. M. (1996). Uncoupling of obesity from insulin resistance through a targeted mutation in aP2, the adipocyte fatty acid binding protein. Science 274 (5291), 1377–1379. 10.1126/science.274.5291.1377 8910278

[B54] Icer M. A. Yıldıran H. (2020). Effects of nutritional status on serum fetuin-A level. Crit. Rev. Food Sci. Nutr. 60 (11), 1938–1946. 10.1080/10408398.2019.1631751 31232084

[B55] Isayama F. Froh M. Yin M. Conzelmann L. O. Milton R. J. McKim S. E. (2004). TNF α‐induced ras activation due to ethanol promotes hepatocyte proliferation independently of liver injury in the mouse. Hepatology 39 (3), 721–731. 10.1002/hep.20137 14999690

[B56] Iwaki M. Matsuda M. Maeda N. Funahashi T. Matsuzawa Y. Makishima M. (2003). Induction of adiponectin, a fat-derived antidiabetic and antiatherogenic factor, by nuclear receptors. Diabetes 52 (7), 1655–1663. 10.2337/diabetes.52.7.1655 12829629

[B57] Ix J. H. Sharma K. (2010). Mechanisms linking obesity, chronic kidney disease, and fatty liver disease: The roles of fetuin-A, adiponectin, and AMPK. J. Am. Soc. Nephrol. 21 (3), 406–412. 10.1681/ASN.2009080820 20150538 PMC4473254

[B58] Jahnen-Dechent W. Heiss A. Schäfer C. Ketteler M. (2011). Fetuin-A regulation of calcified matrix metabolism. Circ. Res. 108 (12), 1494–1509. 10.1161/CIRCRESAHA.110.234260 21659653

[B59] Jersmann H. P. Dransfield I. Hart S. P. (2003). Fetuin/alpha2-HS glycoprotein enhances phagocytosis of apoptotic cells and macropinocytosis by human macrophages. Clin. Sci. 105 (3), 273–278. 10.1042/CS20030126 12725640

[B60] Kaess B. M. Enserro D. M. McManus D. D. Xanthakis V. Chen M. H. Sullivan L. M. (2012). Cardiometabolic correlates and heritability of fetuin-A, retinol-binding protein 4, and fatty-acid binding protein 4 in the Framingham Heart Study. J. Clin. Endocrinol. Metab. 97 (10), E1943–E1947. 10.1210/jc.2012-1458 22855337 PMC3674297

[B61] Karmilin K. Schmitz C. Kuske M. Korschgen H. Olf M. Meyer K. (2019). Mammalian plasma fetuin-B is a selective inhibitor of ovastacin and meprin metalloproteinases. Sci. Rep. 9 (1), 546. 10.1038/s41598-018-37024-5 30679641 PMC6346019

[B62] Kaushik S. V. Plaisance E. P. Kim T. Huang E. Y. Mahurin A. J. Grandjean P. W. (2009). Extended‐release niacin decreases serum fetuin‐A concentrations in individuals with metabolic syndrome. Diabetes. Metab. Res. Rev. 25 (5), 427–434. 10.1002/dmrr.967 19405044

[B63] Kellermann J. Haupt H. Auerswald E. Muller-Ester W. (1989). The arrangement of disulfide loops in human α2-HS glycoprotein: Similarity to the disulfide bridge structures of cystatins and kininogens. J. Biol. Chem. 264 (24), 14121–14128. 10.1016/s0021-9258(18)71651-7 2760061

[B64] Kelley D. E. McKolanis T. M. Hegazi R. A. Kuller L. H. Kalhan S. C. (2003). Fatty liver in type 2 diabetes mellitus: Relation to regional adiposity, fatty acids, and insulin resistance. Am. J. Physiol. Endocrinol. Metab. 285 (4), E906–E916. 10.1152/ajpendo.00117.2003 12959938

[B65] Kettler M. Bongartz P. Westenfeld R. (2003). Association of low fetuin-A (AHSG) concentration in serum with cardiovascular mortality in patients on dialysis: A cross-sectional study. Lancet 361 (9360), 827–833. 12642050 10.1016/S0140-6736(03)12710-9

[B66] Kim H-E. Choi S-E. Lee S-J. Lee J. H. Lee Y. J. Kang S. S. (2008). Tumour necrosis factor-alpha-induced glucose-stimulated insulin secretion inhibition in INS-1 cells is ascribed to a reduction of the glucose-stimulated Ca2+ influx. J. Endocrinol. 198 (3), 549–560. 10.1677/JOE-08-0131 18593820

[B67] Komsa-Penkova R. S. Golemanov G. M. Radionova Z. V. Tonchev P. T. Iliev S. D. Penkov V. V. (2017). Fetuin-A–alpha2-heremans-schmid glycoprotein: From structure to a novel marker of chronic diseases part 1. Fetuin-A as a calcium chaperone and inflammatory marker. J. Biomed. Clin. Res. 10 (2), 90–97. 10.1515/jbcr-2017-0015

[B68] Kübler D. Gosenca D. Wind M. Heid H. Friedberg I. Jahnen-Dechent W. (2007). Proteolytic processing by matrix metalloproteinases and phosphorylation by protein kinase CK2 of fetuin-A, the major globulin of fetal calf serum. Biochimie 89 (3), 410–418. 10.1016/j.biochi.2006.10.012 17110014

[B69] Kumbla L. Bhadra S. Subbiah M. (1991). Multifunctional role for fetuin (fetal protein) in lipid transport. FASEB J. 5 (14), 2971–2975. 10.1096/fasebj.5.14.1721594 1721594

[B70] Kundranda M. N. Henderson M. Carter K. J. Gorden L. Binhazim A. Ray S. (2005). The serum glycoprotein fetuin-A promotes Lewis lung carcinoma tumorigenesis via adhesive-dependent and adhesive-independent mechanisms. Cancer Res. 65 (2), 499–506. 10.1158/0008-5472.499.65.2 15695392

[B71] Kuśnierz-Cabala B. Gurda-Duda A. Panek J. Fedak D. Dumnicka P. Solnica B. (2010). Serum fetuin A concentrations in patients with acute pancreatitis. Clin. Lab. 56 (5-6), 191–195. 20575466

[B72] Lanuza-Masdeu J. Arévalo M. I. Vila C. Barbera A. Gomis R. Caelles C. (2013). *In vivo* JNK activation in pancreatic β-cells leads to glucose intolerance caused by insulin resistance in pancreas. Diabetes 62 (7), 2308–2317. 10.2337/db12-1097 23349497 PMC3712047

[B73] Laughlin G. A. McEvoy L. K. Barrett‐Connor E. Daniels L. B. Ix J. H. (2014). Fetuin‐A, a new vascular biomarker of cognitive decline in older adults. Clin. Endocrinol. 81 (1), 134–140. 10.1111/cen.12382 PMC4053483 24325554

[B74] Lee C-C. Bowman B. H. Yang F. M. (1987). Human alpha 2-HS-glycoprotein: The A and B chains with a connecting sequence are encoded by a single mRNA transcript. Proc. Natl. Acad. Sci. U. S. A. 84 (13), 4403–4407. 10.1073/pnas.84.13.4403 3474608 PMC305097

[B75] Leite-Browning M. L. McCawley L. J. Choi O. H. Matrisian L. M. Ochieng J. (2002). Interactions of alpha2-HS-glycoprotein (fetuin) with MMP-3 and murine squamous cell carcinoma cells. Int. J. Oncol. 21 (5), 965–971. 12370742

[B76] Leite-Browning M. L. McCAWLEY L. J. Jahnen-Dechent W. King L. E. Matrisian L. M. Ochieng J. (2004). Alpha 2-HS glycoprotein (fetuin-A) modulates murine skin tumorigenesis. Int. J. Oncol. 25 (2), 319–324. 15254728

[B77] Lewis J. Andre C. (1980). Effect of human alpha 2HS glycoprotein on mouse macrophage function. Immunology 39 (3), 317–322. 7439929 PMC1457798

[B78] Lewis J. Andre C. (1981). Enhancement of human monocyte phagocytic function by alpha 2HS glycoprotein. Immunology 42 (3), 481–487. 7203533 PMC1458439

[B79] Li W. Zhu S. Li J. Huang Y. Zhou R. Fan X. (2011). A hepatic protein, fetuin-A, occupies a protective role in lethal systemic inflammation. PloS one 6 (2), e16945. 10.1371/journal.pone.0016945 21347455 PMC3035675

[B80] Lin X. Braymer H. Bray G. York D. A. (1998). Differential expression of insulin receptor tyrosine kinase inhibitor (fetuin) gene in a model of diet-induced obesity. Life Sci. 63 (2), 145–153. 10.1016/s0024-3205(98)00250-1 9674949

[B81] Lin Y-H. Franc V. Heck A. J. (2018). Similar albeit not the same: In-depth analysis of proteoforms of human serum, bovine serum, and recombinant human fetuin. J. Proteome Res. 17 (8), 2861–2869. 10.1021/acs.jproteome.8b00318 29966421 PMC6079914

[B82] Mathews S. T. Singh G. P. Ranalletta M. Cintron V. J. Qiang X. Goustin A. S. (2002). Improved insulin sensitivity and resistance to weight gain in mice null for the Ahsg gene. Diabetes 51 (8), 2450–2458. 10.2337/diabetes.51.8.2450 12145157

[B83] Matsui I. Hamano T. Mikami S. Fujii N. Takabatake Y. Nagasawa Y. (2009). Fully phosphorylated fetuin-A forms a mineral complex in the serum of rats with adenine-induced renal failure. Kidney Int. 75 (9), 915–928. 10.1038/ki.2008.700 19190677

[B84] Mellgren R. L. Huang X. (2007). Fetuin A stabilizes m-calpain and facilitates plasma membrane repair. J. Biol. Chem. 282 (49), 35868–35877. 10.1074/jbc.M706929200 17942392

[B85] Metry G. Stenvinkel P. Qureshi A. Carrero J. J. Yilmaz M. I. Barany P. (2008). Low serum fetuin‐A concentration predicts poor outcome only in the presence of inflammation in prevalent haemodialysis patients. Eur. J. Clin. Investig. 38 (11), 804–811. 10.1111/j.1365-2362.2008.02032.x 19021697

[B86] Moghimi S. M. Hunter A. C. (2001). Recognition by macrophages and liver cells of opsonized phospholipid vesicles and phospholipid headgroups. Pharm. Res. 18 (1), 1–8. 10.1023/a:1011054123304 11336343

[B87] Mori K. Emoto M. Inaba M. (2012). Fetuin-A and the cardiovascular system. Adv. Clin. Chem. 56, 175–195. 10.1016/b978-0-12-394317-0.00010-8 22397032

[B88] Mori K. Emoto M. Inaba M. (2011). Fetuin-A: A multifunctional protein. Recent Pat. Endocr. Metab. Immune Drug Discov. 5 (2), 124–146. 10.2174/187221411799015372 22074587

[B89] Mori K. Emoto M. Yokoyama H. Araki T. Teramura M. Koyama H. (2006). Association of serum fetuin-A with insulin resistance in type 2 diabetic and nondiabetic subjects. Diabetes care 29 (2), 468. 10.2337/diacare.29.02.06.dc05-1484 16443916

[B90] Mukhopadhyay S. Mondal S. A. Kumar M. Dutta D. (2014). Proinflammatory and antiinflammatory attributes of fetuin-a: A novel hepatokine modulating cardiovascular and glycemic outcomes in metabolic syndrome. Endocr. Pract. 20 (12), 1345–1351. 10.4158/EP14421.RA 25370330

[B91] Mukhuty A. Fouzder C. Kundu R. (2021). Blocking TLR4-NF-κB pathway protects mouse islets from the combinatorial impact of high fat and fetuin-A mediated dysfunction and restores ability for insulin secretion. Mol. Cell. Endocrinol. 532, 111314. 10.1016/j.mce.2021.111314 33989718

[B92] Mukhuty A. Fouzder C. Kundu R. (2021). Fetuin-A secretion from β-cells leads to accumulation of macrophages in islets, aggravates inflammation and impairs insulin secretion. J. Cell. Sci. 134 (21), jcs258507. 10.1242/jcs.258507 34643217

[B93] Mukhuty A. Fouzder C. Kundu R. (2022). Fetuin‐A excess expression amplifies lipid induced apoptosis and β‐cell damage. J. Cell. Physiol. 237 (1), 532–550. 10.1002/jcp.30499 34224584

[B94] Mukhuty A. Fouzder C. Mukherjee S. Malick C. Mukhopadhyay S. Bhattacharya S. (2017). Palmitate induced Fetuin-A secretion from pancreatic β-cells adversely affects its function and elicits inflammation. Biochem. Biophys. Res. Commun. 491 (4), 1118–1124. 10.1016/j.bbrc.2017.08.022 28797566

[B95] Nackiewicz D. Dan M. He W. Kim R. Salmi A. Rutti S. (2014). TLR2/6 and TLR4-activated macrophages contribute to islet inflammation and impair beta cell insulin gene expression via IL-1 and IL-6. Diabetologia 57 (8), 1645–1654. 10.1007/s00125-014-3249-1 24816367

[B96] Nakamura O. Kazi J. A. Ohnishi T. ArakakiN. Shao Q. Kajihara T. (1999). Effects of rat fetuin on stimulation of bone resorption in the presence of parathyroid hormone. Biosci. Biotechnol. Biochem. 63 (8), 1383–1391. 10.1271/bbb.63.1383 10500999

[B97] Nie Z. (1992). Fetuin: Its enigmatic property of growth promotion. Am. J. Physiol. 263 (3), C551–C562. 10.1152/ajpcell.1992.263.3.C551 1384344

[B98] Ochi A. Mori K. Emoto M. Nakatani S. Morioka T. Motoyama K. (2014). Direct inhibitory effects of pioglitazone on hepatic fetuin-A expression. PLoS One 9 (2), e88704. 10.1371/journal.pone.0088704 24551137 PMC3923806

[B99] Ochieng J. Nangami G. Sakwe A. Moye C. Alvarez J. Whalen D. (2018). Impact of Fetuin-A (AHSG) on tumor progression and type 2 diabetes. Int. J. Mol. Sci. 19 (8), 2211. 10.3390/ijms19082211 PMC6121429 30060600

[B100] Okamoto T. Tsutaya C. Hatakeyama S. Konishi S. Okita K. Tanaka Y. (2018). Low serum butyrylcholinesterase is independently related to low fetuin-A in patients on hemodialysis: A cross-sectional study. Int. Urol. Nephrol. 50 (9), 1713–1720. 10.1007/s11255-018-1957-z 30128921

[B101] Olivier E. Soury E. Ruminy P. Husson A. ParmentierF. DaveauM. (2000). Fetuin-B, a second member of the fetuin family in mammals. Biochem. J. 350 (2), 589–597. 10.1042/bj3500589 10947975 PMC1221288

[B102] Ombrellino M. Wang H. Yang H. ZhangM. Vishnubhakat J. FrAzier A. (2001). Fetuin, a negative acute phase protein, attenuates TNF synthesis and the innate inflammatory response to carrageenan. Shock (Augusta, Ga) 15 (3), 181–185. 10.1097/00024382-200115030-00004 11236900

[B103] Öner-İyidoğan Y. Kocak H. Seyidhanoğlu M. Gurdol F. Gulcubuk A. Yildirim F. (2013). Curcumin prevents liver fat accumulation and serum fetuin-A increase in rats fed a high-fat diet. J. Physiol. Biochem. 69 (4), 677–686. 10.1007/s13105-013-0244-9 23430567

[B104] Osawa M. Umetsu K. Sato M. Ohki T. YukawaN. Suzuki T. (1997). Structure of the gene encoding human alpha 2-HS glycoprotein (AHSG). Gene 196 (1-2), 121–125. 10.1016/s0378-1119(97)00216-3 9322749

[B105] Ou H-Y. Yang Y-C. Wu H-T. Wu J. S. Lu F. H. Chang C. J. (2012). Increased fetuin-A concentrations in impaired glucose tolerance with or without nonalcoholic fatty liver disease, but not impaired fasting glucose. J. Clin. Endocrinol. Metab. 97 (12), 4717–4723. 10.1210/jc.2012-2414 23066121

[B106] Pal D. Dasgupta S. Kundu R. Maitra S. Das G. Mukhopadhyay S. (2012). Fetuin-A acts as an endogenous ligand of TLR4 to promote lipid-induced insulin resistance. Nat. Med. 18 (8), 1279–1285. 10.1038/nm.2851 22842477

[B107] Pedersen K. O. (1944). Fetuin, a new globulin isolated from serum. Nature 154 (3914), 575. 10.1038/154575a0

[B108] Ray S. Lukyanov P. Ochieng J. (2003). Members of the cystatin superfamily interact with MMP-9 and protect it from autolytic degradation without affecting its gelatinolytic activities. Biochim. Biophys. Acta 1652 (2), 91–102. 10.1016/j.bbapap.2003.08.004 14644044

[B109] Reinehr T. Roth C. L. (2008). Fetuin-A and its relation to metabolic syndrome and fatty liver disease in obese children before and after weight loss. J. Clin. Endocrinol. Metab. 93 (11), 4479–4485. 10.1210/jc.2008-1505 18728159

[B110] Reynolds J. L. Joannides A. J. Skepper J. N. McNair R. Schurgers L. J. Proudfoot D. (2004). Human vascular smooth muscle cells undergo vesicle-mediated calcification in response to changes in extracellular calcium and phosphate concentrations: A potential mechanism for accelerated vascular calcification in ESRD. J. Am. Soc. Nephrol. 15 (11), 2857–2867. 10.1097/01.ASN.0000141960.01035.28 15504939

[B111] Reynolds J. L. Skepper J. N. McNair R. Kasama T. Gupta K. Weissberg P. L. (2005). Multifunctional roles for serum protein fetuin-a in inhibition of human vascular smooth muscle cell calcification. J. Am. Soc. Nephrol. 16 (10), 2920–2930. 10.1681/ASN.2004100895 16093453

[B112] Richardson S. Willcox A. Bone A. Morgan N. G. (2009). Islet-associated macrophages in type 2 diabetes. Diabetologia 52 (8), 1686–1688. 10.1007/s00125-009-1410-z 19504085

[B113] Rochette C. N. Rosenfeldt S. Heiss A. Narayanan T. Ballauff M. Jahnen-Dechent W. (2009). A shielding topology stabilizes the early stage protein–mineral complexes of fetuin‐A and calcium phosphate: A time‐resolved small‐angle X‐ray study. Chembiochem 10 (4), 735–740. 10.1002/cbic.200800719 19222044

[B114] Rudloff S. Janot M. Rodriguez S. Dessalle K. Jahnen-Dechent W. Huynh-Do U. (2021). Fetuin-A is a HIF target that safeguards tissue integrity during hypoxic stress. Nat. Commun. 12 (1), 549. 10.1038/s41467-020-20832-7 33483479 PMC7822914

[B115] Samocha-Bonet D. Tam C. S. Campbell L. V. Heilbronn L. K. (2014). Raised circulating fetuin-A after 28-day overfeeding in healthy humans. Diabetes Care 37 (1), e15–e16. 10.2337/dc13-1728 24356603

[B116] Sardana O. Goyal R. Bedi O. (2021). Molecular and pathobiological involvement of fetuin-A in the pathogenesis of NAFLD. Inflammopharmacology 29 (4), 1061–1074. 10.1007/s10787-021-00837-4 34185201

[B117] Sato H. Kazama J. J. Wada Y. Kuroda T. Narita I. Gejyo F. (2007). Decreased levels of circulating alpha2-Heremans-Schmid glycoprotein/Fetuin-A (AHSG) in patients with rheumatoid arthritis. Intern. Med. 46 (20), 1685–1691. 10.2169/internalmedicine.46.6269 17938521

[B118] Seyithanoğlu M. Öner-İyidoğan Y. Doğru-Abbasoğlu S. Tanrikulu-Kucuk S. Kocak H. Beyhan-Ozdas S. (2016). The effect of dietary curcumin and capsaicin on hepatic fetuin-A expression and fat accumulation in rats fed on a high-fat diet. Arch. Physiol. Biochem. 122 (2), 94–102. 10.3109/13813455.2015.1120753 26706937

[B119] Shen X. Yang L. Yan S. Zheng H. Liang L. Cai X. (2015). Fetuin A promotes lipotoxicity in β cells through the TLR4 signaling pathway and the role of pioglitazone in anti-lipotoxicity. Mol. Cell. Endocrinol. 412, 1–11. 10.1016/j.mce.2015.05.014 25986658

[B120] Silvestri L. Pagani A. Nai A. De Domenico I. Kaplan J. Camaschella C. (2008). The serine protease matriptase-2 (TMPRSS6) inhibits hepcidin activation by cleaving membrane hemojuvelin. Cell. Metab. 8 (6), 502–511. 10.1016/j.cmet.2008.09.012 18976966 PMC2648389

[B121] Singh M. Sharma P. K. Garg V. K. Mondal S. C. Singh A. K. Kumar N. (2012). Role of fetuin-A in atherosclerosis associated with diabetic patients. J. Pharm. Pharmacol. 64 (12), 1703–1708. 10.1111/j.2042-7158.2012.01561.x 23146032

[B122] Song A. Xu M. Bi Y. Xu Y. Huang Y. Li M. (2011). Serum fetuin-A associates with type 2 diabetes and insulin resistance in Chinese adults. PLoS One 6 (4), e19228. 10.1371/journal.pone.0019228 21556362 PMC3083420

[B123] Stefan N. Fritsche A. Weikert C. Boeing H. Joost H. G. Haring H. U. (2008). Plasma fetuin-A levels and the risk of type 2 diabetes. Diabetes 57 (10), 2762–2767. 10.2337/db08-0538 18633113 PMC2551687

[B124] Stefan N. Hennige A. M. Staiger H. Machann J. Schick F. Krober S. M. (2006). Alpha2-Heremans-Schmid glycoprotein/fetuin-A is associated with insulin resistance and fat accumulation in the liver in humans. Diabetes care 29 (4), 853–857. 10.2337/diacare.29.04.06.dc05-1938 16567827

[B125] Stefan N. Hennige A. M. Staiger H. Machann J. Schick F. Krober S. M. (2006). Alpha2-Heremans-Schmid glycoprotein/fetuin-A is associated with insulin resistance and fat accumulation in the liver in humans. Diabetes care 29 (4), 853–857. 10.2337/diacare.29.04.06.dc05-1938 16567827

[B126] Stirnberg M. Maurer E. Arenz K. Babler A. Jahnen-Dechent W. Gutschow M. (2015). Cell surface serine protease matriptase-2 suppresses fetuin-A/AHSG-mediated induction of hepcidin. Biol. Chem. 396 (1), 81–93. 10.1515/hsz-2014-0120 25205713

[B127] Stöcker W. Karmilin K. Hildebrand A. Westphal H. Yiallouros I. Weiskirchen R. (2014). Mammalian gamete fusion depends on the inhibition of ovastacin by fetuin-B. Biol. Chem. 395 (10), 1195–1199. 10.1515/hsz-2014-0189 25205729

[B128] Sun J. Zhang D. Xu J. Chen C. Deng D. Pan F. (2020). Circulating FABP4, nesfatin-1, and osteocalcin concentrations in women with gestational diabetes mellitus: A meta-analysis. Lipids Health Dis. 19 (1), 199. 10.1186/s12944-020-01365-w 32861247 PMC7456504

[B129] Swallow C. J. Partridge E. A. Macmillan J. C. Tajirian T. DiGuglielmo G. M. Hay K. (2004). alpha2HS-glycoprotein, an antagonist of transforming growth factor beta *in vivo*, inhibits intestinal tumor progression. Cancer Res. 64 (18), 6402–6409. 10.1158/0008-5472.CAN-04-1117 15374947

[B130] Szweras M. Liu D. Partridge E. A. Pawling J. Sukhu B. Clokie C. (2002). Alpha 2-HS glycoprotein/fetuin, a transforming growth factor-beta/bone morphogenetic protein antagonist, regulates postnatal bone growth and remodeling. J. Biol. Chem. 277 (22), 19991–19997. 10.1074/jbc.M112234200 11901155

[B131] Tajirian T. Dennis J. W. Swallow C. J. (2000). Regulation of human monocyte proMMP‐9 production by fetuin, an endogenous TGF‐β antagonist. J. Cell. Physiol. 185 (2), 174–183. 10.1002/1097-4652(200011)185:2<174::AID-JCP2>3.0.CO;2-X 11025439

[B132] Tang Y. L. Jiang J. H. Wang S. Liu Z. Tang X. Q. Peng J. (2015). TLR4/NF-κ B signaling contributes to chronic unpredictable mild stress-induced atherosclerosis in ApoE-/-mice. PloS one 10 (4), e0123685. 10.1371/journal.pone.0123685 25860573 PMC4393302

[B133] Thiele L. Diederichs J. E. Reszka R. Merkle H. P. Walter E. (2003). Competitive adsorption of serum proteins at microparticles affects phagocytosis by dendritic cells. Biomaterials 24 (8), 1409–1418. 10.1016/s0142-9612(02)00525-2 12527282

[B134] Trepanowski J. Mey J. Varady K. (2015). Fetuin-A: A novel link between obesity and related complications. Int. J. Obes. 39 (5), 734–741. 10.1038/ijo.2014.203 25468829

[B135] Van Oss C. Gillman C. Bronson P. Border J. R. (1974). Opsonic properties of human serum alpha-2 hs glycoprotein. Immunol. Commun. 3 (4), 329–335. 10.3109/08820137409061113 4142783

[B136] Wang H. E Sama A. (2012). Anti-inflammatory role of fetuin-A in injury and infection. Curr. Mol. Med. 12 (5), 625–633. 10.2174/156652412800620039 22292896 PMC3349766

[B137] Wang H. Li W. Zhu S. Li J. D'Amore J. Ward M. F. (2010). Peripheral administration of fetuin-A attenuates early cerebral ischemic injury in rats. J. Cereb. Blood Flow. Metab. 30 (3), 493–504. 10.1038/jcbfm.2009.247 19953099 PMC2860738

[B138] Weikert C. Stefan N. Schulze M. B. Pischon T. Berger K. Joost H. G. (2008). Plasma fetuin-a levels and the risk of myocardial infarction and ischemic stroke. Circulation 118 (24), 2555–2562. 10.1161/CIRCULATIONAHA.108.814418 19029462

[B139] Wilson N. L. Schulz B. L. Karlsson N. G. Packer N. H. (2002). Sequential analysis of N-and O-linked glycosylation of 2D-PAGE separated glycoproteins. J. Proteome Res. 1 (6), 521–529. 10.1021/pr025538d 12645620

[B140] Wojtysiak-Duma B. Malecha Jędraszek A. Burska A. (2010). Serum fetuin-A levels in patients with type 2 diabetes mellitus. Ann. UMCS Sect. 1500, 2.

[B141] Xiao Y. Shu L. Wu X. Liu Y. Cheong L. Y. Liao B. (2021). Fatty acid binding protein 4 promotes autoimmune diabetes by recruitment and activation of pancreatic islet macrophages. JCI insight 6 (7), 141814. 10.1172/jci.insight.141814 33690220 PMC8119222

[B142] Yoshioka Y. Gejyo F. Marti T. Rickli E. E. Burgi W. Offner G. D. (1986). The complete amino acid sequence of the A-chain of human plasma alpha 2HS-glycoprotein. J. Biol. Chem. 261 (4), 1665–1676. 10.1016/s0021-9258(17)35992-6 3944104

[B143] Yu C-L. Tsai M-H. (2001). Fetal fetuin selectively induces apoptosis in cancer cell lines and shows anti-cancer activity in tumor animal models. Cancer Lett. 166 (2), 173–184. 10.1016/s0304-3835(01)00417-7 11311490

[B144] Zhao Z-W. Lin C-G. Wu L-Z. Luo Y. K. Fan L. Dong X. F. (2013). Serum fetuin-A levels are associated with the presence and severity of coronary artery disease in patients with type 2 diabetes. Biomarkers 18 (2), 160–164. 10.3109/1354750X.2012.762806 23410047

[B145] Zhou Z-w. Ju H-x. Sun M-z. Chen H. M. Fu Q. P. Jiang D. M. (2018). Serum fetuin-A levels in obese and non-obese subjects with and without type 2 diabetes mellitus. Clin. Chim. Acta. 476, 98–102. 10.1016/j.cca.2017.11.023 29174342

